# Transcriptional landscape of repetitive elements in normal and cancer human cells

**DOI:** 10.1186/1471-2164-15-583

**Published:** 2014-07-11

**Authors:** Steven W Criscione, Yue Zhang, William Thompson, John M Sedivy, Nicola Neretti

**Affiliations:** 1 Department of Molecular Biology, Cell Biology, and Biochemistry, Brown University, Providence, RI 02912 USA; 2 Division of Applied Mathematics, Brown University, Providence, RI 02912 USA; 3 Center for Computational Molecular Biology, Brown University, Providence, RI 02912 USA

**Keywords:** Retrotransposon, Transposable element, Prostate cancer, LINE-1, L1, LTR, HERV, Repetitive element, RNA-seq, ChIP-seq

## Abstract

**Background:**

Repetitive elements comprise at least 55% of the human genome with more recent estimates as high as two-thirds. Most of these elements are retrotransposons, DNA sequences that can insert copies of themselves into new genomic locations by a “copy and paste” mechanism. These mobile genetic elements play important roles in shaping genomes during evolution, and have been implicated in the etiology of many human diseases. Despite their abundance and diversity, few studies investigated the regulation of endogenous retrotransposons at the genome-wide scale, primarily because of the technical difficulties of uniquely mapping high-throughput sequencing reads to repetitive DNA.

**Results:**

Here we develop a new computational method called *RepEnrich* to study genome-wide transcriptional regulation of repetitive elements. We show that many of the Long Terminal Repeat retrotransposons in humans are transcriptionally active in a cell line-specific manner. Cancer cell lines display increased RNA Polymerase II binding to retrotransposons than cell lines derived from normal tissue. Consistent with increased transcriptional activity of retrotransposons in cancer cells we found significantly higher levels of L1 retrotransposon RNA expression in prostate tumors compared to normal-matched controls.

**Conclusions:**

Our results support increased transcription of retrotransposons in transformed cells, which may explain the somatic retrotransposition events recently reported in several types of cancers.

**Electronic Supplementary Material:**

Supplementary material is available for this article at 10.1186/1471-2164-15-583 and is accessible for authorized users.

## Background

The initial sequencing of the human genome revealed that ~55% of the genome is comprised of repetitive DNA sequences [1]. More recent computational approaches indicate the proportion of repetitive elements in the human genome may be as high as two-thirds [2]. Identified repetitive DNA sequences can be characterized using five broad categories. Four minor categories, accounting for ~10% of genomic DNA, include simple sequence repeats, segmental duplications, tandem repeats and satellite DNA sequences, and processed pseudogenes. The fifth category is transposable elements, accounting for ~45% of genomic DNA and is primarily composed of retrotransposons. Retrotransposable elements (RTEs) are parasitic DNA sequences that can proliferate by a “copy and paste” mechanism and insert themselves into new genomic positions. RTEs are classified into Long Terminal Repeat (LTR) elements, whose structure and mechanism of retrotransposition resembles that of retroviruses, and non-LTR elements, which do not contain LTRs, resemble integrated mRNAs, and have a distinct mechanism of retrotransposition [1]. In humans only the non-LTR elements are believed to be capable of retrotransposition, and can be classified as either Long Interspersed Nuclear Elements (LINEs) or Short Interspersed Nuclear Elements (SINEs) [3]. They are predominantly represented by the L1 and Alu families, respectively. The process of retrotransposition requires the transcription of an mRNA intermediate and its reverse transcription into cDNA, and can lead to the disruption of genes by insertional mutagenesis. Retrotransposition occurs *de novo* in the germ-line and can cause single-gene mutations that result in disease, an example being hemophilia A [4]. The L1 protein machinery may also retrotranspose copies of genes and structural non-coding RNAs yielding processed pseudogenes.

The majority of our understanding of retrotransposon transcription and function comes from studies of single elements and their DNA sequence, primarily autonomous elements capable of active retrotransposition such as the L1Hs retrotransposon (a human-specific L1 subfamily) or non-autonomous elements such as Alu that can retrotranspose *in trans* using the L1 protein machinery. These studies revealed that endogenous retrotransposons are repressed in human cells under normal conditions, predominantly via silencing by promoter DNA methylation [5]. However, when retrotransposons are expressed, such as in response to cellular stress, Alu is thought to be transcribed by RNA polymerase III (Pol III), and L1 by RNA polymerase II (Pol II) from an internal promoter [5].

Few studies have attempted to survey transposable element transcription genome-wide. High throughput sequencing data poses a challenge to these studies due to the ambiguity in assigning short reads mapping to more than one genomic location (referred to here as multi-mapping reads). Application-specific strategies have been developed to recover multi-mapping reads, such as assignment of Cap Analysis Gene Expression (CAGE) reads to the most represented Transcriptional Start Site (TSS) in CAGE sequencing data [6], a method to identify TSS. A genome-wide analysis of retrotransposon expression using CAGE data revealed that repetitive elements are expressed in the mouse in a tissue-specific manner [7].

More recent attempts to address systematically the ambiguity in read assignment have followed two complementary strategies. The first attempts to include multi-mapping reads in computing the read coverage across the genome by either assigning reads proportionally to all matching regions [8, 9], or by assigning them probabilistically to a specific location based on the local genomic tag context [10]. The second strategy addresses the ambiguity in read mapping by assigning them to subfamilies of repetitive elements as opposed to their specific locations across the genome. Early examples estimated repetitive element enrichment by mapping short read data to consensus sequences [11, 12]. However, this approach did not account for the majority of genomic instances, many of which deviate from the consensus sequence. A more recent example of the second approach incorporated both consensus and genomic instances in the analysis but excluded reads aligning to more than a single repetitive element subfamily [13]. Because individual repetitive element subfamilies are highly conserved within their families, this latter approach excluded a significant fraction of mapping reads from the analysis. For example, the L1PA2 and L1PA3 subfamilies have a high degree of homology; many reads mapping to one of these two subfamilies also map to the other and would be excluded.

In this study we extend these approaches to quantify repetitive element enrichment by utilizing all mapping reads in estimating read counts. The resulting computational pipeline, *RepEnrich*, was integrated with existing computational tools to test for differential enrichment between two or more experimental conditions. We report here the results of a whole-genome analysis of the transcription and regulation of repetitive elements, obtained by applying *RepEnrich* to both RNA-seq and ChIP-seq datasets for RNA Pol II, Pol III and associated transcription factors in a panel of human cell lines, as well as several chromatin activation and repression marks [14–20]. Finally, we identify transposable elements overexpressed in tumor tissue collected from prostate cancer patients [21].

## Results

### Comprehensive assessment of repetitive element enrichment

**Figure 1:**
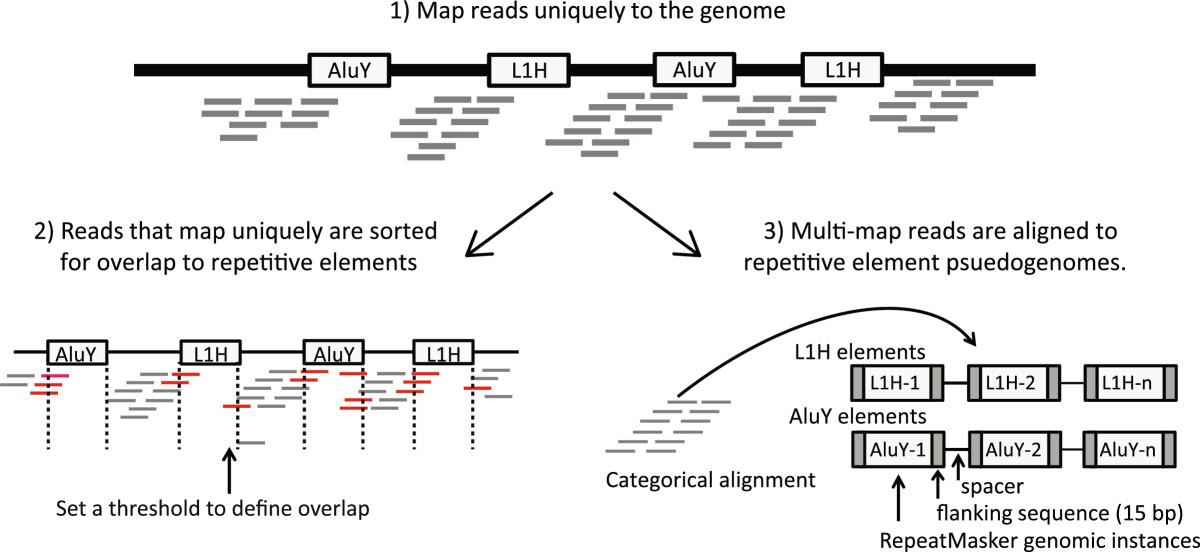
***RepEnrich*** **read mapping strategy.** Reads are mapped to the genome using the *Bowtie1* aligner. Reads mapping uniquely to the genome are assigned to subfamilies of repetitive elements based on their degree of overlap to *RepeatMasker* annotated genomic instances of each repetitive element subfamily. Reads mapping to multiple locations are separately mapped to repetitive element assemblies – referred to as repetitive element psuedogenomes – built from *RepeatMasker* annotated genomic instances of repetitive element subfamilies.

In *RepEnrich*, reads are initially aligned to the unmasked genome and divided into uniquely mapping and multi-mapping reads. Uniquely mapping reads are tested for overlap with repetitive elements, while multi-mapping reads are separately aligned to repetitive element assemblies representing individual repetitive element subfamilies (Figure 1). Repetitive element assemblies are represented by all genomic instances (assembled from the *RepeatMasker* annotation) of an individual repetitive element subfamily, including flanking genomic sequences, concatenated with spacer sequences to avoid spurious mapping of reads spanning multiple instances. The repetitive element assemblies are an extension of the strategy used by Day *et al.*[13], which however only used reads that could be unambiguously assigned to an individual subfamily.

By combining the counts from uniquely mapping reads and multi-mapping reads *RepEnrich* keeps track of all repetitive elements that every read aligns to and systematically estimates enrichment from all mapping reads. Using this strategy we can compute read abundance in three different ways. First, we can compute the total number of reads mapping to each repetitive element subfamily (Additional file 1: Figure S1A), which we refer to as *total counts*. Second, we can compute the total number of reads mapping exclusively to a single repetitive element subfamily. This methodology is similar to the one used Day *et al.* and we refer to it as *unique counts* (Additional file 1: Figure S1B). Third, we can count reads that map to a single repetitive element subfamily assembly once and assign reads that map to multiple subfamilies using a fractional value 1/N_s_ (where N_s_ is the number of repetitive element subfamily assemblies the read maps to), which we call *fractional counts* (Additional file 1: Figure S1C).

To investigate how these three counting strategies differed in their ability to estimate read abundance, we used *in silico* generated ChIP-seq data. The ChIP-seq data simulators currently available [22] cannot modulate the sampling rate of reads at specific loci in the genome. Hence, we developed a general-purpose Hidden Markov Model (HMM) ChIP-seq simulator that can generate sample reads at user-defined emission rates from specified genomic loci. We simulated ChIP-seq and input data in triplicates for whole human chromosomes to represent scenarios in which different families of repetitive elements were enriched. To assess the generality of our results our simulations used different chromosomes, read lengths, and families of enriched repetitive elements (L1, Alu, and SVA). The HMM structure and parameters used in our simulations are described in Additional file 1: Figure S2. Additional file 1: Figure S3 shows a representative read alignment in a 35 kb region of chromosome 19 for a simulation in which retrotransposons in the L1 family were enriched with respect to background.

In all our simulations we applied *RepEnrich* to compute the abundance, expressed in counts per million mapping reads (CPM), for all repetitive elements based on the three counting strategies. Because we knew the exact chromosomal location each read was sampled from in the simulation, we could unambiguously compute the *true abundance* of each repetitive element.

**Figure 2:**
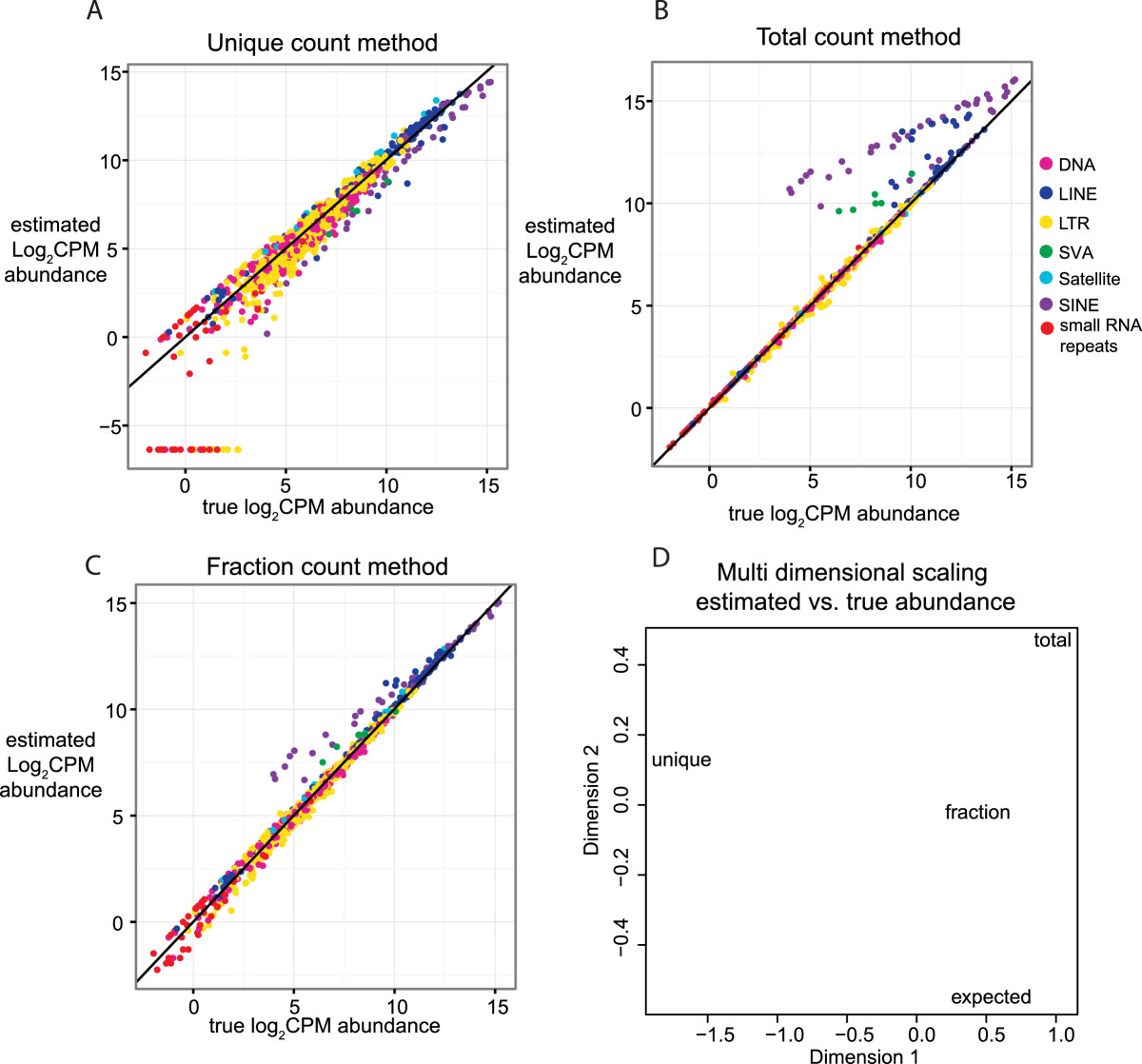
**Performance comparison of counting strategies on simulated L1-enriched data.** Three replicates of ChIP-seq (50 bp single-end reads) data enrichment at L1 elements on chromosome 19 were simulated using the hidden Markov model (HMM) in Additional file 1: Figure S2. The expected average log2CPM for the simulation was computed using the repetitive element counts computed from the true read coordinates. The average log2CPM read abundances, computed by *EdgeR* from *RepEnrich* estimated count values using *total, unique*, and *fractional count* methods were compared to the expected true abundance. The solid line indicates y = x, values falling on the line are identical between the estimated average log2CPM and expected average log2CPM. The repetitive element subfamilies are colored according to class with small RNA repeats including scRNA, rRNA, snRNA, and tRNA classes. **A)** Comparison of the estimated abundance from the *unique count* method, which only sums reads that can be assigned uniquely to a single subfamily of repetitive elements, versus the true abundance. **B)** Comparison of the estimated abundance from the *total count* method, which sums the reads assigned to each repetitive element subfamily and allows for multiple counting of reads, versus the true abundance. **C)** Comparison of the estimated abundance from the *fractional count* method, which sums the reads that fall into each individual repetitive element subfamily once, but adds a fraction for reads mapping to more than one subfamily (1/# of repetitive element sub-families aligned), versus the true abundance. **D)** Multidimensional scaling (MDS) plot of the Euclidean distances between the average log2CPM values for the unique, total, and fractional count estimates of *RepEnrich* and the expected average log2CPM values. The fractional count average log2CPM estimate was closest to the true abundance.

Our simulations revealed clear differences in the performance of the three counting strategies. Figures 2A-C and Additional file 1: Figure S4A-C show the scatterplots of the *RepEnrich* CPM estimate versus the *true abundance* CPM for all repetitive element subfamilies the L1 enrichment and Alu enrichment simulations respectively. The *unique counting* strategy (Figure 2A and Additional file 1: Figure S4A) tends to over- or under-estimate the *true abundance* of repetitive elements and thus introduces the most variance to the estimate. In addition, specific families of repetitive elements show a common bias; most notably SINEs are consistently underestimated. The *total counting* strategy performs better overall but suffers from a strong bias in a few families of repetitive elements, such as SINEs and SINE-Variable Number Tandem Repeat-Alus (SVAs) elements, which are consistently overestimated (Figure 2B and Additional file 1: Figure S4B). The *fractional counting* strategy appears to provide the optimal estimate: deviation from the true abundance is smallest for all subfamilies (Figure 2C and Additional file 1: Figure S4C). The largest deviations occurred for elements with smaller CPM values. Although some of the family-specific biases in the *total counts* are still present, they are greatly reduced and limited to elements with low CPM values.

We confirmed these observations by conducting three additional comparative analyses. First we applied multi-dimensional scaling to the four vectors containing the *unique, total, fractional* and *true* CPMs respectively (Figure 2D and Additional file 1: Figure S4D). The *fractional count* strategy is more similar to the *true abundance* as demonstrated by the smaller distance between these two in the multi-dimensional scaling plot.

Next, we computed the deviation from the 45° line in the scatterplots of estimated abundance vs. true abundance (Additional file 1: Figures S5B-D and S6B-D). Additional file 1: Figures S5A and S6A show the R-squared value for all elements combined and for each repetitive element class separately in two sets of L1 enrichment simulations in with 2 M reads for two different chromosomes. The R-squared values in the *fractional count* strategy were consistently close to 1 only in the case of the fractional count, and varied widely between 0 and 1 for the *unique counts* strategy. Comparison with the scatterplots in Figure 2, which were obtained from simulating 20 M reads, also indicate that the *unique count* strategy is more affected by read coverage than the other two methods and performs poorly at lower coverage.

Finally, we assessed the ability of the various counting methods to reveal significant differences between two experimental samples. To do so we compared the SVA, L1, and Alu enriched samples to their input samples via a Generalized Linear Model (GLM) fit to a negative binomial distribution (see Methods). We created a benchmark set of differentially enriched elements in each simulation by applying the GLM model to the *true abundance* counts, and compared this set to the elements detected as differentially enriched in each of the three counting strategies. In all simulations the *fractional counting* method recovered numerous significant repetitive elements that were identified to be differentially enriched in the *true abundance* comparison benchmark; it also returned the least number of false positive (Additional file 1: Figures S7 and S8).

To assure our observations were not restricted to *in silico* data we compared the performance of the *fractional counting* and *unique counting* methods on real ChIP-seq data. We utilized a ChIP-seq dataset for RNA polymerase II (Pol II) conducted in K562 cell-line (Additional file 2: Table S1), and applied the GLM to identify repetitive elements enrichment for Pol-II with respect to input. Consistently with our simulations, the *fractional counting* method identified more elements as enriched for Pol-II with respect to the *unique counting* method (Additional file 1: Figure S9).

Because the *fractional counts* displayed the least bias and variance in the estimation of repetitive element abundance, and was most similar to the *true abundance,* we chose to use *fractional counts* as the default counting strategy for *RepEnrich*.

### Experimental design

To investigate transcription of different classes of repetitive elements in human cells we applied *RepEnrich* to a collection of publicly available RNA-seq and ChIP-seq datasets. We collected high-throughput sequencing data from the ENCyclopedia Of DNA Elements (ENCODE), the Gene Expression Omnibus (GEO) and the European Nucleotide Archive (ENA). A detailed list of individual samples can be found in Additional file 2: Table S1.

Transcription in eukaryotes is performed by three different RNA Polymerases, Pol I-III. With the exception of Pol I, which specializes in ribosomal RNA (rRNA) transcription, both Pol II and III are known to transcribe repetitive elements [5, 23]. Hence, to address the question of how repetitive elements are transcribed we utilized ChIP-seq data for Pol II, Pol III, and TFIIIB (a Pol III-associated transcription factor complex). ChIP-seq for TFIIIB subunits has previously been used as additional support of Pol III binding, because TFIIIB is necessary for Pol III promoter recognition [15, 24]. For Pol II, we analyzed ChIP datasets generated with a Pol II antibody that does not distinguish active and inactive enzymes, as well as an antibody to Pol II phosphorylated on serine 2 (Pol II S2), which is specific for the active elongating enzyme. To our knowledge, no ChIP-seq dataset for RNA Pol I is currently available.

We adopted a comprehensive approach that included the analysis of not only transposable elements, but also other classes of repetitive elements annotated within *RepeatMasker*, with the exclusion of simple sequence repeats. Among the repetitive element classes we examined for Pol II and Pol III binding were the small structural RNAs and their processed pseudogenes. Small structural RNAs including tRNAs, snRNAs, and rRNAs are included in the *Repeatmasker* annotation because of the high degree of sequence homology to processed psuedogenes. Previous Pol III ChIP-seq studies indicated that the tRNA pseudogenes are occupied by Pol III [24], which is not surprising since tRNA Pol III promoters are internal. Some Pol II transcribed snRNA psuedogenes may also be transcribed, and have been found to be associated with L1-encoded proteins [25].

To investigate the transcription and regulation of repetitive elements in a variety of cell types, we collected data from multiple cell lines. Specifically, our analysis included Pol II and III ChIP-seq performed with IMR-90 fibroblasts, K562 chronic myelogenous leukemia (CML) cells, HeLa adenocarcinoma cells and GM12878 lymphoblastoid cells, as well as additional RNA Pol II-only ChIP-seq data for HUVEC (human umbilical vein) endothelial cells and peripheral blood-derived erythroblast cells (PBDE) purified from human blood samples (Additional file 2: Table S1) [15–17, 24]. K562 and HeLa are cancer-derived transformed cell lines, GM12878 is an EBV-immortalized cell line, and IMR-90, HUVEC and PDBE are normal (non-immortalized) cells.

### Regulation and transcription of repetitive elements in human cells

**Figure 3:**
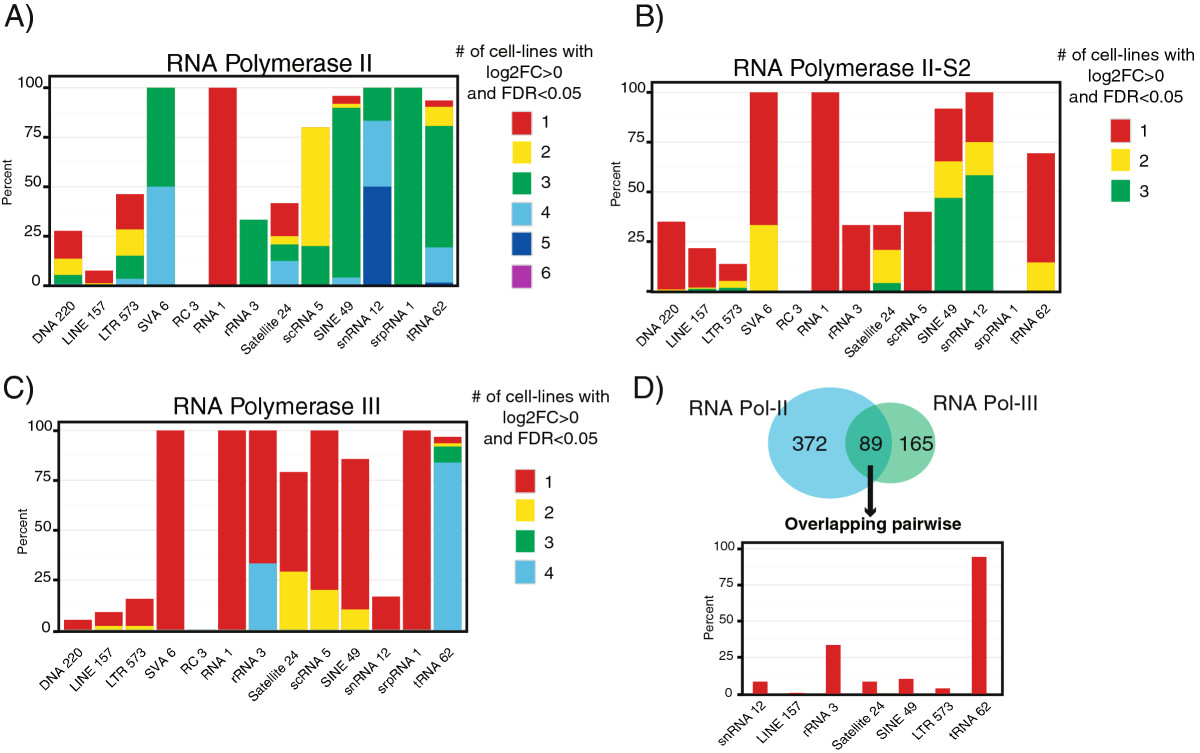
**RNA polymerase binding patterns to repetitive elements. A)** RNA Pol II, **B)** active RNA Pol II S2, and **C)** RNA Pol III were assessed for binding to repetitive elements using generalized linear model (GLM) comparisons of ChIP versus input. To view the binding patterns we examined percent of repetitive element sub-families for the major classes of repetitive elements that displayed significant (FDR <0.05) positive enrichment (Log2FC >0). The color-coding corresponds to the number of cell lines that displayed the significant positive enrichment. The x-axis labels the class of repetitive element and the adjacent number indicates how many repetitive element sub-families fall within that class. **D)** The repetitive elements that displayed significant (FDR <0.05) positive enrichment (Log2FC >0) for RNA Pol II and RNA Pol III were compared for overlap across the same cell line. The 89 repetitive elements that displayed co-enrichment within the same cell line for RNA Pol II and RNA Pol III were then examined for representation of the major classes of repetitive elements, expressed as a percent.

All ChIP-seq and RNA-seq data were processed with *RepEnrich* to generate counts for all repetitive element subfamilies. Log2-fold-changes between ChIP and input samples as well as the statistical significance were then evaluated using a generalized linear model (GLM) fit to a negative binomial distribution (see Methods).The repetitive elements that displayed the most shared pattern of Pol II binding between the cell lines were the snRNAs (Figure 3A, B). This is consistent with the known universal role of snRNAs in RNA processing and their transcription by Pol II (with the exception of the U6 snRNA, which is a Pol III transcript). Likewise, we observed ubiquitous Pol III binding to tRNAs and the 5S rRNA across all the cell lines examined (Figure 3C). Transposable elements rarely displayed consistency across all cell lines, and instead primarily displayed significant Pol II or Pol III binding in one or a few cell lines (Figure 3). This is at least partially explained by a tendency of retrotransposable elements to be expressed more highly in transformed versus normal (non-transformed) cell lines.

One interesting feature we identified was significant co-occupancy of Pol II and Pol III at some repetitive elements. When overlapped within the same cell line, 89 repetitive element subfamilies were co-occupied by Pol II and Pol III (Figure 3D). The majority of these repetitive elements were tRNAs (Figure 3D). Because tRNAs are short and the reads near their borders map uniquely to the genome, we could examine tRNA elements in the genome browser for further evidence of Pol II and Pol III co-occupancy. We identified multiple instances where Pol II and Pol III were bound at or near the same tRNA gene (Additional file 1: Figure S10 A), and instances where snRNA genes, including Pol III transcribed U6, were co-occupied by Pol II and Pol III (Additional file 1: Figure S10 B).

To further characterize the transcription of repetitive elements, we examined binding of Pol III-specific transcription factors, as well as RNA transcript subcellular localization and polyadenylation status. The tRNA transcriptional signature displayed evidence of Pol III transcription from a type II promoter (Additional file 1: Figure S11, S12). As expected, the tRNA transcripts were localized to the cytosol and not polyadenylated (Additional file 1: Figure S12). Satellite repeat sequences displayed predominantly Pol II binding, although some subfamilies also displayed Pol III binding (Additional file 1: Figure S12). Satellite RNAs were predominantly nuclear and polyadenylated, consistent with being primarily Pol II transcripts (Additional file 1: Figure S12). The majority of transposable elements did not display strong transcriptional signatures (Additional file 1: Figure S12, S13). Most notably, LINE retrotransposons, the major active class of retrotransposons in humans, displayed very few subfamilies with significant binding of Pol II or Pol III (Additional file 1: Figure S13). DNA transposable elements, which are believed to be inactive in the human genome, also displayed few subfamilies with Pol II or Pol III enrichment (Additional file 1: Figure S13).

SINE elements, predominantly represented by Alu subfamilies, displayed some genome-wide enrichment for Pol II and III binding; the Pol II binding may be due to the high representation of Alus within gene introns (Additional file 1: Figure S12). Similar to Canella *et al.* we observed significant binding of Pol III to SINE elements, likely representing independent SINE transcription [15]. SINE RNAs displayed a cytosolic and non-polyadenylated enrichment pattern, which is consistent with SINE elements being transcribed from internal Pol III promoters (Additional file 1: Figure S12) [5]. By far the most transcriptionally active endogenous retrotransposons we observed were in the LTR family (Additional file 1: Figure S13). Many LTR elements displayed significant binding by Pol II, and some also displayed enrichment for Pol III (Figure 3, Additional file 1: Figure S13). As noted above, the majority of LTR retrotransposon subfamilies that displayed polymerase binding did so in one or a few cell lines.

### The endogenous retrovirus HERV-Fc1 is actively transcribed by Pol II in a CML cell line

**Figure 4:**
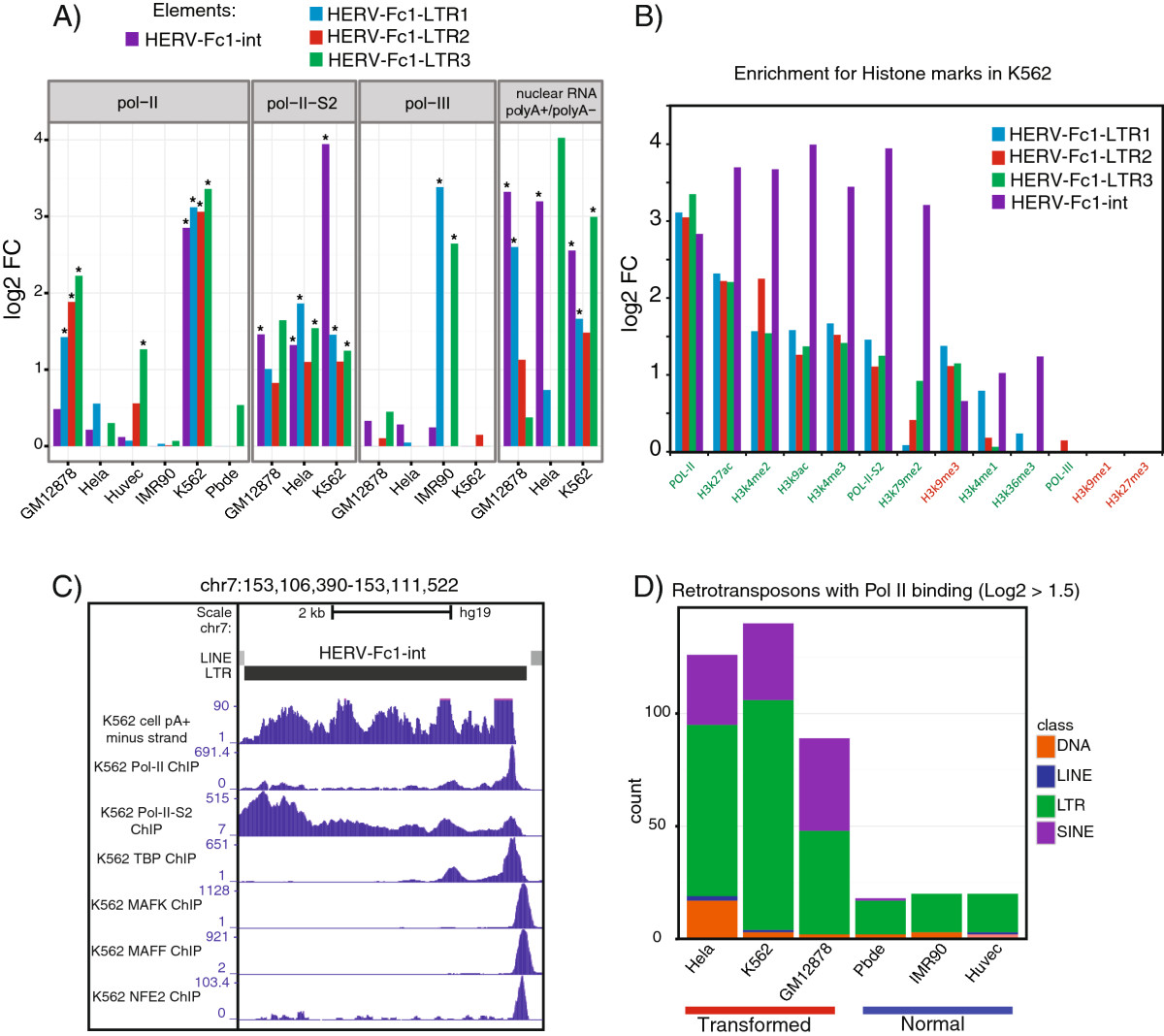
**HERV-Fc1 and Pol II binding in transformed vs. normal cell lines.** LTR and other transposable elements displayed differences in RNA Pol II binding in transformed versus normal cell lines. **A** **)** The LTR subfamily HERV-FC1 displayed cell line specific transcriptional profiles for the LTRs (LTR1-3) or internal region (int) of HERV-FC1. The GLM results are plotted as log2FCs for Pol II enrichment and differential RNA-seq analysis. The differential RNA-seq analysis compares the PolyA + vs. PolyA – enrichment of Nuclear RNA (positive log2FC values indicates PolyA + enrichment). **B** **)** The enrichment of ChIP compared to input for RNA Pol II, active RNA Pol II-S2, active marks of transcription (H3K27ac, H3K4me2, H3K9ac, H3K4me3, H3K79me2, H3K4me1, H3K36me2) and repressed heterochromatin (H3K9me1, H3K9me3, H3K27me3) for the LTRs (LTR1-3) or internal region (int) of HERV-FC1. **C** **)** Genome browser view of the primary locus of HERV-FC1-int contributing to expression in the K562 cell line. The ENCODE signal tracks for K562 cell PolyA + RNA (minus strand), RNA Pol II ChIP, RNA Pol II-S2 ChIP, TBP ChIP, MAFK ChIP, MAFF ChIP, and NFE2 ChIP were visualized on chr7. All other cell lines for which there was cell PolyA + RNA available displayed minimal signal at this locus. **D** **)** The count of transposable elements displaying modest positive enrichment, log2FC >1.5, in transformed versus normal cell lines. The counts are colored by the class of transposable element.

Among the LTR elements, numerous elements displayed Pol II enrichment that was significant in at least one cell-line (FDR < 0.05, for a full list see Additional file 1: Figure S14). One element that displayed a particularly striking binding was the internal portion of HERV-Fc1, most prominently in K562 CML cell-line. We chose to focus on K562 cell-line for additional analysis because the internal region of HERV-Fc1 displayed 7- and 15-fold enrichment for Pol II and Pol II S2 in this cell-line (Figure 4A). To further examine the behavior of HERV-Fc1 in K562 cells we applied *RepEnrich* to ENCODE ChIP-seq data for histone marks associated with active euchromatin (H3K27ac, H3K4me2, H3K9ac, H3K4me3, H3K79me2, H3K4me1, H3K36me2) and repressed heterochromatin (H3K9me1, H3K9me3, H3K27me3). We found that the HERV-Fc1 element, especially its internal region, was highly enriched for marks associated with active transcription and depleted for marks associated with repression (Figure 4B). These results indicate derepression of the HERV-Fc1 retrotransposon in the K562 CML cell line.The HERV-Fc1 subfamily is represented by few copies in the human genome, and its internal region, HERV-Fc1-int, has only seven copies in the hg19 build. We therefore examined all the genomic loci of HERV-Fc1 in K562 cells using the UCSC genome browser and ENCODE tracks. A single HERV-Fc1 internal element on chromosome 7 displayed RNA expression from the minus strand in K562 cells but not in any other ENCODE cell line for which PolyA + RNA-seq is available (Figure 4C). This region also displayed binding for Pol II and active Pol II-S2 as well as the TATA-box binding protein (TBP). We also noted the binding of MAFK, MAFF, and NFE2 transcription factors at the promoter of the HERV-Fc1 element.

### L1 retrotransposons are significantly overexpressed in prostate tumor tissue

Somatic retrotransposition events were recently reported in several cancers [26]. We therefore examined normal and transformed cell lines for Pol II binding and tested whether transformed cells displayed more permissive binding of Pol II to retrotransposons. Our results indicated that a larger number of transposable elements show at least 1.5-fold enrichment for Pol II in HeLa, K562, and GM12878 transformed cells than in PBDE, IMR90, and HUVEC normal cells (Figure 4D). This is especially true for LTR retrotransposons. Hierarchical clustering of Pol II binding for LTR elements with significant enrichment in at least one cell line revealed that normal cell-lines clustered separately from cancer and transformed cells (Additional file 1: Figure S14). We thus wanted to investigate further whether the increased Pol II binding in transformed cell lines contributed to increased expression of transposable elements. However, in this dataset the transformed and normal cells were derived from a variety of tissues and hence direct comparison of retrotransposon transcription was not possible.

**Figure 5:**
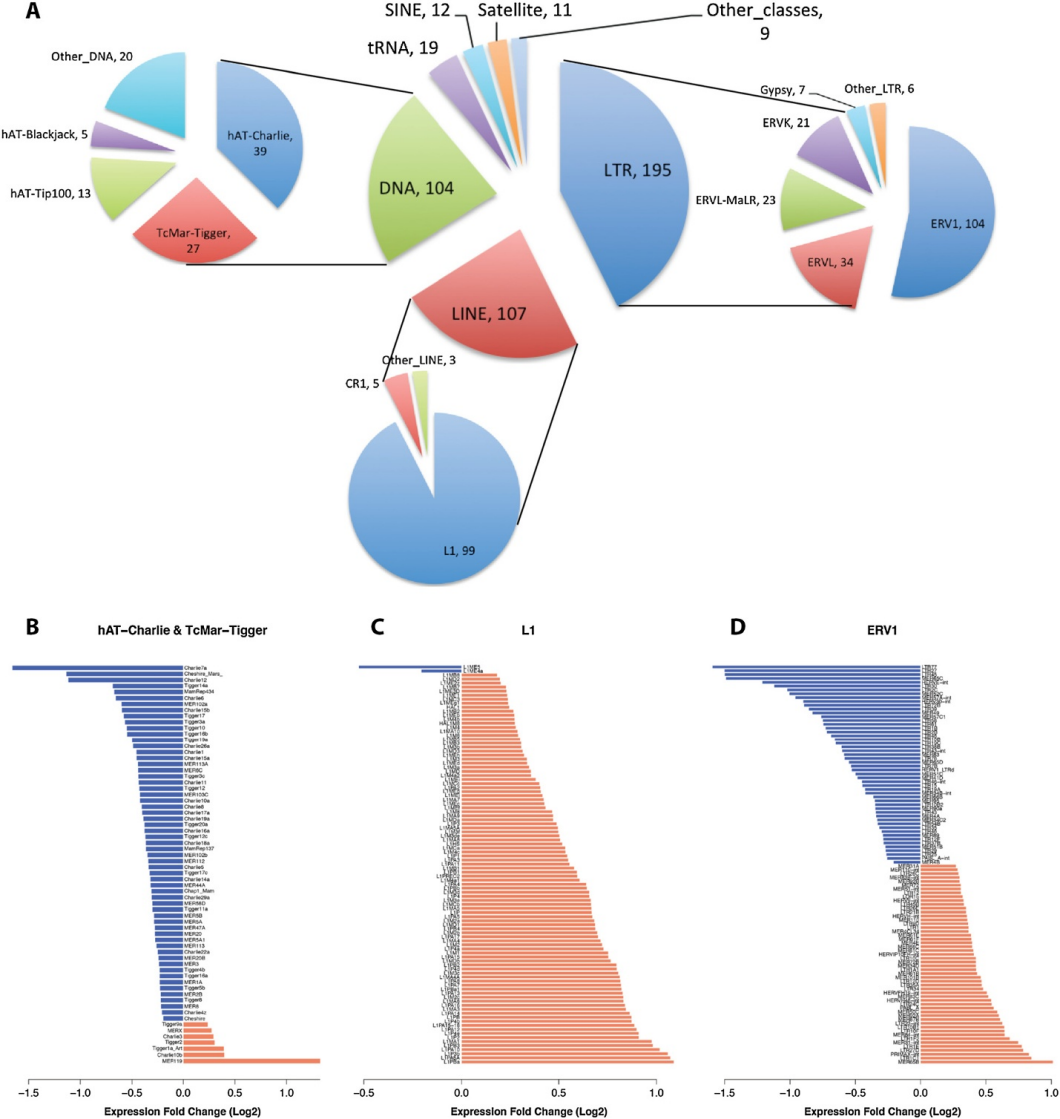
**Repetitive elements differentially expressed in prostate cancer tissue.** **(** **A** **)** Classes and families of repetitive elements differentially expressed in prostate cancer tumor tissue versus normal tissue. The number next to each class and family name corresponds to the number of differentially expressed subfamilies (FDR < 0.05). **(** **B** **-** **D** **)** Expression fold-change between prostate cancer tumor tissue and normal tissue computed by the GLM on the 14 patients. The most represented family of DNA, LINE and LTR elements are shown.

To better control for individual and tissue-specific expression differences, we tested our hypothesis using a RNA-seq tumor dataset that contains data for matched prostate tumor and normal tissue from 14 patients with different grades of prostate cancer [21]. We detected 475 retrotransposon subfamilies that exhibited significant differential expression in tumor tissue (FDR < 0.05), prevalently from the LTR, LINE and DNA classes (Figure 5A). Interestingly, very few SINE subfamilies were differentially expressed in prostate tumor versus normal tissue. Most of the LTR subfamilies were endogenous retroviruses, with ERV1 being the most represented. Of the ERV1 family, 53 subfamilies were overexpressed and 51 subfamilies were under-expressed (Figure 5D). Most of the differentially expressed DNA elements belonged to the hAT-Charlie and TcMar-Tigger families, and the vast majority of them (59 out of 66) were significantly under-represented in tumor tissue (Figure 5B). For LINEs, 99 out of 107 subfamilies belonged to the L1 family of retrotransposons, and the vast majority of these (97 out of 99) were overexpressed in tumor tissue (Figure 4C).

**Figure 6:**
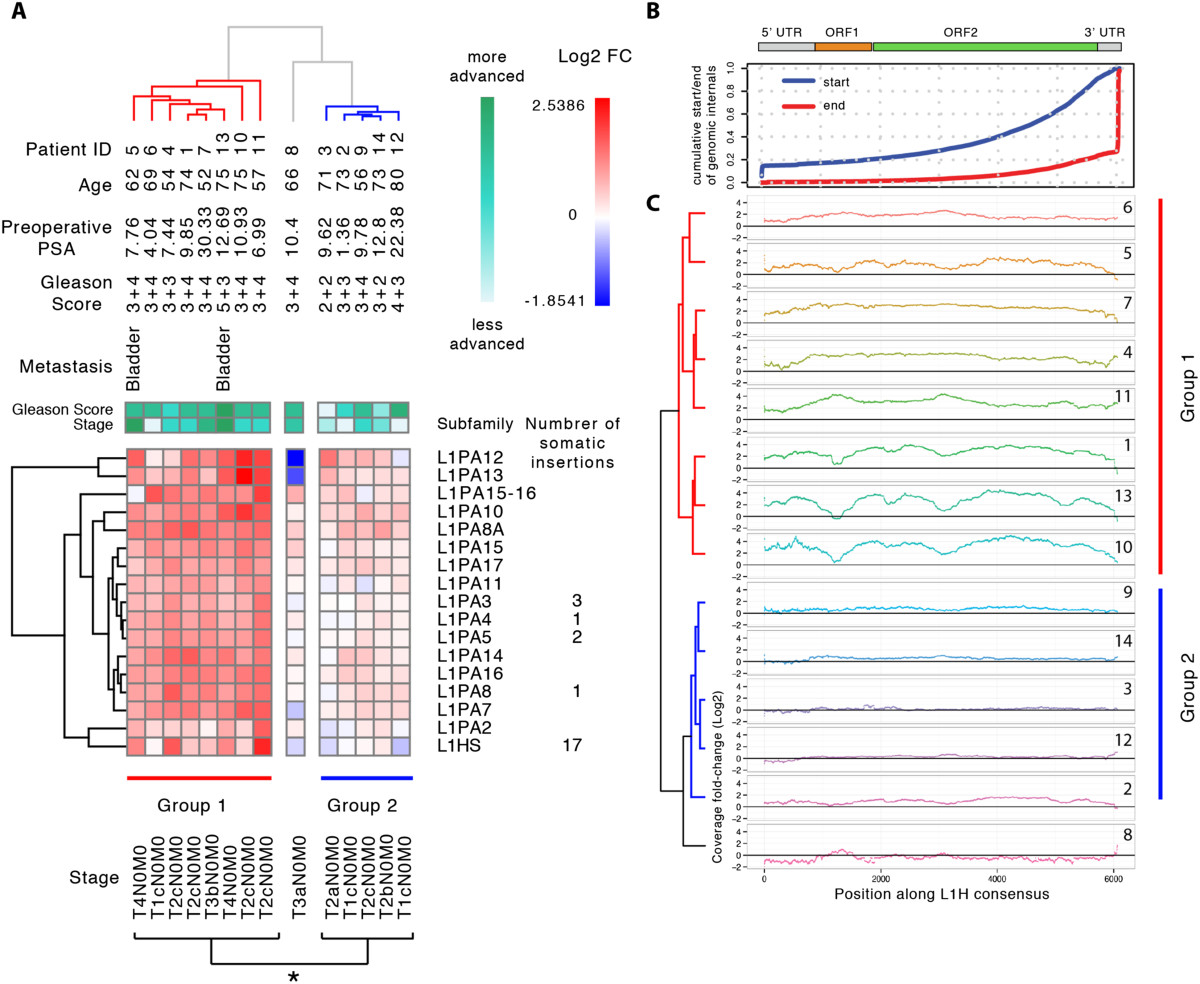
**Primate-specific L1 elements are overexpressed in a subclass of patients with more advanced tumor progression. (A)** Clustering of log2 expression fold-changes in the subset of primate specific L1s that showed significant differential expression reveals two major classes of patients (Group 1 and Group 2). Group 1 shows widespread overexpression of primate specific L1s and contains patients with more advanced tumor progression. The number of somatic insertions refers to the number of previously reported somatic retrotransposition events for that L1 subfamily identified in prostate cancer [26]. **(B)** All L1 sequences in the human genome were fetched and mapped to L1Hs consensus using permissive, local alignment parameters to analyze data. Using this distribution we computed the cumulative distribution of start and end positions of genomic L1s with respect to the consensus to describe the background distribution of L1s that can potentially map to the consensus element. **(C)** Coverage of L1 sequences in prostate tumor versus normal RNA-seq that map to L1Hs consensus using a local alignment (Bowtie2). The log2FC was computed for each position along the L1Hs consensus from tumor and normal-matched RNA-seq coverage. Hierarchical clustering was done based on the log2FC using Euclidean metrics.

L1 LINEs are the most active retrotransposons in humans and their retrotransposition was recently documented in multiple cancers [26–28]. Figure 6A shows a heatmap of the log2 fold changes between tumor and normal tissue for evolutionarily recent primate and human-specific L1 subfamilies that displayed statistically significant differences. We applied bi-clustering (see Figure legend) and identified two major groups of patients. Group 1 showed a marked overexpression of the primate-specific L1s, while group 2 showed a lower level of overexpression and in some cases underrepresentation. Patient 8 appeared to be an outlier. We studied the association of these two groups with some clinical parameters available for each patient [21]. We detected no association with patient age or preoperative PSA, but a significant association with the stage of the cancer: group 1 patients showed a more advanced cancer state with respect to group 2, as defined by the TNM score (p = 0.04, Mann Whitney U test; Figure 6A).

Interestingly, all the novel somatic retrotransposition events identified in prostate cancer [26] belonged to the sub-families of L1s that displayed significant enrichment in our dataset (Figure 6A). In particular, 17 of them were from the human-specific L1Hs subfamily. Hence, we examined the L1Hs elements more closely by mapping all RNA-seq reads to the L1Hs consensus using Bowtie2 local alignment mode. This method is not entirely specific to L1Hs as closely homologous L1PA elements are also represented. The L1Hs subfamily and its closely related primate-specific L1PA subfamilies are composed of genomic instances that are 3′ biased as a consequence of a 5′ truncation that frequently occurs during retrotransposition [29] (Figure 6B, top panel). The fold-change in coverage along the L1Hs consensus between tumor and normal tissue was increased 2- to 4-fold across the entire length of the element, including the 5′ UTR region in patient group 1. This is consistent with transcription of elements in the genome that are full length or close to full length. We also observed interesting and conserved patterns of fold-changes. For example, patients 1, 10 and 13 in group 1 show dipping at 4 locations corresponding to L1 ORF1 and ORF2, while patient 11 in the same group displayed the opposite behavior.

Many repetitive element insertions, including those of L1 and Alu [30], are found in the introns of genes. The starting material for most RNA-seq libraries is poly-A purified total cellular RNA, which is predominantly mature mRNA that is free of introns. However, a small fraction of total cellular RNA is composed of pre-mRNA, also known as heterogeneous nuclear RNA (hnRNA), which also contains intronic sequences and can be polyadenylated. Hence, some of the reads assigned to repetitive elements could have originated from this small hnRNA pool. To address this we examined separately the mapping of unique L1Hs and L1PA reads to intronic and intergenic regions, and found very similar tumor-associated increases in the abundance corresponding to both regions (Additional file 1: Figure S15). Hence, the increased transcription of L1 elements in prostate tumors appears to affect equivalently elements inserted outside of known genes, and those inserted within introns.

## Discussion

The majority of the human genome is comprised of repetitive sequences, most of which are represented by parasitic retrotransposon elements. Recent years have seen increased interest in understanding their regulation because of the important roles in genome evolution, development, and disease [31–35]. A prolific expansion of sequencing data, combined with new experimental and computational methods in genomics and transcriptomics, have spurred an extensive exploration of chromatin regulation, and the temporal and spatial organization of the RNA transcriptome. In spite of these new technologies, fundamental computational obstacles remain for the analysis of repetitive elements in the short-read data produced by high-throughput sequencing. This is because short reads of repetitive elements align ambiguously and cannot be assigned to unique locations in the genome.

We wanted to develop a computational pipeline to estimate enrichment and differential expression of repetitive elements in ChIP-seq and RNA-seq datasets. Because signal from repetitive elements in many cases is likely to be weaker than from genes, as a consequence of their low level of activity, we favored a strategy that assigned reads to repetitive element subfamilies as opposed to individual instances. Previous work excluded reads that map to more than one repetitive element subfamily [13]. This approach can be problematic, because some individual elements are highly conserved. For example, multiple sequence alignment of the consensus sequence for primate specific L1s reveals a high degree of homology between individual elements, despite the fact that these consensus sequences represent distinct repetitive element subfamilies (Additional file 1: Figure S16). Many multi-mapping reads tend to align with multiple repetitive element subfamilies (Additional file 1: Figure S17). Our tests of this counting strategy indicated that exclusion of reads mapping to more than one repetitive element subfamily would exclude 64% of 30 bp repetitive mapping reads and 51% of 50 bp repetitive mapping reads (Additional file 1: Figure S17). Furthermore, requiring unambiguous assignment of reads to individual subfamilies will introduce a bias towards less conserved repeats, which will be assigned relatively higher counts.

To assess how to optimally count reads that map to more than one subfamily, we used *in silico* ChIP-seq data simulations where the *true abundance* of repetitive elements was known. Double counting reads mapping to multiple subfamilies (*total counting* approach) tended to overestimate enrichment of Alu and SVA elements, while excluding those same reads (the *unique counting* method used by Day *et al.*) introduced a similar bias but in the opposite direction, as well as a larger variance in the count estimate (Figure 2A). These biases are likely a consequence of the high degree of sequence homology between subfamilies, and are particularly evident in the Alu and SVA families. Alu emerged relatively recently in primate evolution (~60 million years ago), and thus displays a high degree of sequence homology between subfamilies [36]. SVA elements are also highly homologous as they arose even more recently in hominid evolution [37]. A third counting strategy, based on assigning fractional values to each read mapping to multiple subfamilies (*fractional counting* approach), reduced both the bias and variance of the estimate. It most closely approximates the *true abundance*, and recovers more differentially enriched elements in both simulated and real data. Hence we selected *fractional counting* as the optimal strategy.

Based on this analysis we developed a new computational pipeline, *RepEnrich*, for genome-wide studies of repetitive elements in ChIP-seq and RNA-seq high-throughput data. Our methodology extends existing strategies by utilizing all mappable reads in estimating read counts. *RepEnrich* is a flexible pipeline that can readily incorporate different sequence aligners, multiple sequencing data types, and can easily interface with existing statistical packages for downstream analysis. We demonstrate the utility of *RepEnrich* here by examining a large collection of high-throughput datasets to analyze transcriptional regulation of repetitive elements in multiple cell lines and human tissues.

*RepEnrich* readily documented, in a genome-wide manner, several known aspects of the transcriptional activity of repetitive elements, especially small structural non-coding RNAs such as tRNAs, snRNAs, and rRNAs. As expected, tRNAs were predominantly transcribed by Pol III from a type II promoter and were predominantly enriched in the non-polyadenylated fraction and in the cytosol. The snRNAs were observed to be bound by Pol II, in agreement with their known transcriptional mechanism. One interesting observation was that many small structural non-coding RNAs, especially tRNAs, displayed co-occupancy of binding by Pol II and Pol III (Figure 3D). While the co-binding of Pol II and Pol III to small structural non-coding RNAs has been described previously at specific genomic locations [17], our results suggests such association occurs genome-wide.

Polymerase binding to small structural non-coding RNA elements was observed to be wide-spread across all the cell lines examined, which is consistent with their core roles in basic biological processes. Very low levels of polymerase enrichment were found at LINEs and DNA transposons, which is likely a consequence of their constitutive repression by DNA methylation and heterochromatin silencing mechanisms [5]. SINEs and LTR elements showed significant polymerase binding that was typically restricted to one or a few of the cell lines examined (Figure 3A, B and C). The LTR subfamilies were the most active retrotransposable elements, with a general trend towards increased polymerase binding in transformed cells (Figure 4D).

Although LTR retrotransposons are thought to be mostly inactive in humans, and very few cases of novel germ-line and somatic retrotranspositions have been reported [5, 26], our results are consistent with recent genome-wide studies of chromatin accessibility. Analysis of DNase I hypersensitive sites (DHS), markers of accessible chromatin, revealed many cell line-specific changes mapping to retrotransposable elements [38]. In particular, LTR retrotransposons displayed the majority of DHS changes, many of which correlated with changes in chromatin accessibility. Evidence has also emerged that LTR elements might function as enhancers [39, 40]. Similarly, our results suggest that LTR retrotransposons are bound by RNA polymerases and are transcribed in a cell line-specific manner.

Among the LTR elements with Pol II binding, the endogenous retrovirus HERV-Fc1 displayed a large-degree of Pol II and Pol II S2 enrichment with the most prominent binding in a K562 CML line. Active Pol II transcription was also supported by RNA-seq enrichment in the polyadenylated fraction, as well as enrichment of several chromatin activation marks in ChIP-seq data. Although the HERV super-family of retrotransposons is not thought to be active for retrotransposition, several of its members have been associated with multiple diseases. For example, increased transcription of HERV-K family members has been reported in amyotrophic lateral sclerosis (ALS) [25], CML [41], and recently in multiple sclerosis (MS) [42]. Seven HERV-Fc1 elements are currently annotated, and we were able to identify a single genomic locus representing the source of most of the ChIP-seq and RNA-seq signal. Interestingly, at this locus we detected enrichment for binding of the TATA-box binding protein (TBP), as well as the MAFK, MAFF, and NFE2 transcription factors. The MAF family transcription factors contain mutations that are associated with CML [43] and heterodimerize with NFE2 [8]. These binding sites might be exposed due to loss of silencing at repetitive genomic regions in the K562 cancer cell line, consistent with evidence that loss of DNA methylation can strongly activate HERV-Fc1 [44].

One striking result of our analysis was that transformed cell lines consistently displayed a wider pattern of Pol II enrichment than normal cells (Figure 4D). A recent report on genome-wide changes in chromatin accessibility in embryonic stem cells (ESC), differentiated cells, and cancer cells may shed some light on our observations [45]. As ESCs differentiate into various cell types, the proportion of shared DHSs decreases, however, cancer cells gain back many of the DHSs originally found in ESCs. It was suggested that cancer cells adopt a more accessible chromatin landscape, similar to ESCs. Although this particular study did not look specifically at retrotransposons, combining this model with our results on Pol II binding in transformed cells suggests that genomic regions harboring transposable elements might be globally de-repressed and increase their transcriptional activity in cancer.

To further examine the transcriptional activity of retrotransposons in cancer, we examined RNA-seq data from prostate tumors [21]. Many repetitive element families were differentially expressed in prostate tumors, with most of the changes occurring within LINE, LTR, and DNA class elements. LINE elements displayed a striking tendency to be upregulated in prostate tumors. A closer look at L1 regulation revealed that patients could be separated into two groups based on their transcriptional profiles (Figure 6A). We found that patients in group 1 showed higher levels of L1 expression in their tumors and, on average, were diagnosed with a more advanced stage of cancer. Recently, novel somatic retrotransposition events have been identified in several different cancers, including ovarian, prostate, hepatocellular, and colon [26–28]. The majority of these new events involved evolutionarily recent human-specific L1Hs, primate-specific L1PA and Alu elements. For prostate cancer, 26 out of 28 new retrotranspositions identified [26] belonged to the L1Hs and L1PA families that were also significantly upregulated in our analysis. Because only full-length elements are competent for retrotransposition and the majority of L1Hs elements in the genome are 5′ truncated, we further studied changes in read coverage along the entire consensus L1Hs sequence. We found that tumors of group 1 patients showed a 2-fold (or greater) increase in read coverage and that read coverage was elevated equivalently across the entire element including the 5′ end. This suggests that the increase in transcription involved predominantly full-length elements and was initiated at the L1 promoter.

## Conclusions

In summary, our study underscores the richness of information on the transcriptional regulation of repetitive elements, and transposable elements in particular, contained in publically available, high throughput sequencing datasets. Because the amount of this information is expected to vastly increase in the near future, dedicated computational pipelines, such as *RepEnrich*, will be of great utility in mining these datasets. *RepEnrich* allows for the analysis of repetitive elements in any organism with a reference genome available that has repetitive element annotation (such as Repeatmasker annotation). *RepEnrich* also allows for a custom repetitive element annotation, which can be used for a variety of applications where multi-mapping reads become an issue such as gene clusters repeats that appear in tandem duplicates.

Our study also supports the importance of activation of endogenous retrotransposons as an important, and probably universal, feature of cancer. Whether retrotransposable elements are drivers or passengers of the cancer development process is still an open question and will require further investigation. In addition, we suggest that they will have considerable utility as biomarkers, and in combination with other genomic features, will help in elucidating cancer subtypes, progression and prognosis.

## Methods

### Analysis of repetitive element enrichment using *RepEnrich*

Sample reads were aligned to the genome using Bowtie1 with the requirement that reads map uniquely, *command = bowtie hg19 -p 16 -t -m 1 -S --chunkmbs 512 --max multimap.fastq input.fastq output.sam*[46]. Reads mapping to multiple locations of the genome were assigned to a separate FASTQ file (i.e*. --max*). Annotation was constructed from *RepeatMasker* annotated genomic instances of repetitive elements (downloaded from Repeatmasker.org). The genomic coordinates of repetitive elements were used to build repetitive element psuedogenome assemblies for each distinct repetitive element subfamilies. Repetitive element psuedogenome assemblies were built by concatenating genomic instances of each repetitive element subfamily, their flanking genomic sequences (default = 15 bp), and a spacer sequence (default = 200 bp) in FASTA format, in a manner similar to Day *et al.*[13]. These psuedogenomes were indexed using Bowtie. A genomic feature file was also built in BED format, which describes the coordinates of all annotated repetitive element instances. The genomic feature files in BED format and the distinct repetitive element psuedogenome assemblies in FASTA format were used to separately analyze the unique mapping reads and the reads mapping to more than one location. Reads mapping to unique genomic positions were sorted based on overlap with repetitive element genomic instances. To conduct the overlap we used Bedtools to intersect the alignment file and the genomic instances of repetitive elements [47]. Reads that map to more than one location are categorically aligned to the repetitive psuedogenome assemblies using Bowtie. For paired-end reads, each mate pair is separately mapped to the repetitive psuedogenome assemblies. *RepEnrich* systematically tracks all repetitive element subfamilies a given read aligns for all reads. We can determine the number of reads mapping to repetitive element subfamilies, repetitive element families, or repetitive element classes. *RepEnrich* uses three separate ways of classifying the reads that map to multiple repetitive element subfamilies: *total counts, unique counts*, and *fractional counts*. The *total counts* output sums all reads that map to an individual repetitive element subfamily. The *unique counts* output sums only reads that can be uniquely assigned to a single repetitive element subfamily, similar to the output of Day *et. al.*[13]. The *fractional counts* sums reads mapping uniquely to a repetitive element subfamily once and counts reads mapping to multiple subfamilies using a fraction 1/N_s_, where N_s_ = number of repetitive element subfamilies the read aligns with. The *fractional count* rounds the estimate for a subfamily to the nearest integer and is the default method used by *RepEnrich.*

### Availability

The *RepEnrich* tutorial and source code is available for download at our github repository https://github.com/nerettilab/RepEnrich. *RepEnrich* supports analysis for ChIP-seq and RNA-seq for any organism where a reference genome and repetitive element annotation (such as Repeatmasker annotation) is available. *RepEnrich* also supports custom repetitive element or repeat feature annotation in bed format.

### Simulation of ChIP-seq datasets

To conduct the ChIP-seq simulation we developed a hidden Markov model (HMM) that simulates separate states for different genomic features over the length of a chromosome. The strategy is similar to approaches used for previous studies addressing ChIP-seq simulation, however, we extended these methods to cover an entire chromosome and to use underlying information about genomic features [22]. The output for the HMM is the probability that a read is selected from a given genomic position in a ChIP-seq experiment. This probability is derived from the emission state profile generated by the HMM. The transition matrix for the HMM simulates whether a given base pair along the length of the chromosome is in a high or low emission state. The simulator was built such that differential enrichment profiles could be generated by defining the coordinates of repetitive elements, or other genomic features. To simulate enrichment over a repetitive element, we specified a transition state probability matrix that yielded more frequent occupancy of the high emission state for their coordinates. The output for the simulation is the true start positions of all the simulated reads. We then generated reads from the start positions in FASTA format.

We used the ChIP-seq simulation to evaluate the predictive power of RepEnrich. To test the repetitive element analysis we simulated ChIP-seq data on human chromosomes 5, 10, and 19 (see Additional file 1: Figure S2 for HMM parameters). For the simulation of separate chromosomes we used only RepeatMasker genomic instances present on human chromosomes we examined (build hg19). We simulated ChIP-seq data for three experimental comparisons and six experimental conditions. We examined conditions where L1, Alu, and SVA family retrotransposons were enriched and conditions where the L1, Alu, and SVA family retrotransposons were near background, considered an input. Each condition was simulated in triplicate with a parameter to introduce technical variance. For chromosome 19 we simulated a situation with high sequencing depth (twenty million reads) at three read lengths (30, 50, and 100 base pairs). For chromosomes 5 and 10 we simulated a situation with lower sequencing depth (two million reads) at three read lengths (35, 50, and 75 base pairs). Simulated reads were aligned uniquely to human chromosome 19 and reads mapping to multiple locations were output to a separate FASTA file. Repetitive element enrichment was determined by *RepEnrich*. The expected abundance of repetitive element enrichment was determined for the various conditions using the true position of the simulated reads. The simulated positions of the reads were also used to generate the true alignment file, in bam format, as if all the multi-mapping reads had mapped uniquely. Using the true positions the expected count for each repetitive element subfamily was determined by overlapping the reads with the genomic coordinates of each repetitive element subfamily using Bedtools [47].

Using the read counts determined by *RepEnrich, fractional, unique*, and *total counting* methods and the expected count we calculated the normalized read abundance or CPM and conducted differential enrichment analysis. To do so, the various count estimates generated by RepEnrich were analyzed using EdgeR bioconductor package for statistically significant enrichment of repetitive elements in simulated ChIP-seq conditions [48]. EdgeR uses a generalized linear model (GLM) to identify differential enrichment by fitting the genomic count data to a negative binomial distribution. Recent work extends the use of EdgeR from RNA-seq analysis of differential expression to diverse types of genomic count data arising from ChIP-seq experiments [49]. The data were first normalized using trimmed mean of M-values (TMM) normalization method and manually inputted total mapping reads [50]. Using Edger built-in functions we could then compute the normalized read abundance. EdgeR was then used to make a pooled comparison L1, Alu, SVA enriched samples versus input samples, where L1, Alu, SVA were at background levels (see EdgeR tutorial for pooled comparisons). EdgeR analysis yielded the log2 fold changes for ChIP with respect to input and an associated p-value for each repetitive element subfamily. The p values were corrected using an FDR correction using the method described by Storey et al. [51].

### Analysis of ENCODE ChIP-seq datasets for enrichment to repetitive elements

Raw data for RNA Polymerase ChIP-seq experiments was downloaded in FASTQ from the ENCODE data consortium or the European Nucleotide Archive (for complete list see Additional file 2: Table S1) [14–17, 24, 52]. TFIIIB factor components Bdp1, Brf1, Brf2, and SNAP45 ChIP-seq data was obtained from ENCODE and published datasets [15–17]. K562 ChIP-seq data for active and repressed chromatin marks was downloaded from ENCODE data consortium [53]. ChIP-seq and input samples were mapped uniquely to the genome (build hg19) using Bowtie1 short read aligner [46]. Repetitive element analysis was conducted as described above using RepEnrich software. The *fractional count* output of RepEnrich was used for analysis of RNA Pol II and III ChIP-seq data. The raw fractional counts generated by RepEnrich for RNA polymerases in human cell lines was analyzed using EdgeR bioconductor package for statistically significant enrichment of repetitive elements in ChIP-seq samples with respect to input [48]. The data was first normalized using TMM normalization method and manually inputted total mapping reads [50]. EdgeR was then used to make a pooled comparison between RNA Polymerases ChIP-seq versus input using cell line as an independent factor. EdgeR analysis yielded the log2 fold changes for ChIP with respect to input and an associated FDR value.

### Detecting transcripts from repetitive elements in ENCODE RNA-seq experiments

The RepEnrich method was extended to the analysis of repetitive element reads present in RNA-seq data. Three cell lines were chosen to complement the analysis of RNA polymerases and TFIIIB subunits: GM12878, HeLa, and K562 cells. The RNA-seq data for GM12878, HeLa, and K562 cells was generated as part of the ENCODE project [18–20, 54]. The data includes three sub-cellular compartments including total RNA, cytosol, and nucleus. For each cellular compartment we examined PolyA selected and non-PolyA selected RNAs using duplicate samples. The GM12878, Hela, and K562 cells were sequenced using 75 base pair paired-end reads. The analysis serves as an example of how RepEnrich can also be applied to paired-end data. Reads for all samples were trimmed to 50 base pair paired-end, to avoid inconsistency in sequencing quality present at 3′ distal end of reads from different samples. All reads from each RNA-seq sample were mapped uniquely to the human genome (build hg19) using Bowtie1. We used Bowtie1 for the analysis of RNA-seq because repetitive element reads that map specifically to a splice junction may be unreliable and highly ambiguous. By using Bowtie1 rather than Tophat we simply excluded splice-junction reads from our analysis. The alignments were analyzed using RepEnrich and the fractional count output. Downstream analysis was similar to the analysis of ChIP-seq data using EdgeR, with two key differences. First the manually inputted library sizes were obtained by calculating the total mapping reads of STAR alignment BAM files available through ENCODE data consortium using samtools [48, 55, 56]. To identify significant differences in subcellular compartments we built a GLM in EdgeR and conducted comparisons within K562, HeLa, and GM12878 cell lines between the various compartments (all comparisons described in Additional file 2: Table S1). We decided to treat cell line as a separate factor instead of a covariate due to improved performance of the edgeR GLM model, although both approaches yielded similar results.

### Differential RNA expression analysis of repetitive element subfamilies in prostate cancer

RNA-seq data from 14 prostate tumors and paired normal tissue was analyzed as follows [21]. The 90 bp paired-end RNA-seq reads were mapped uniquely to the human genome (build hg19) using Bowtie1. RepEnrich fractional counts were analyzed using EdgeR as was done for ENCODE RNA-seq data. The published total mapping reads for the study were inputted to EdgeR. To identify repetitive element subfamilies with significant differences in tumor versus control we built a paired GLM in EdgeR using individual as a covariate. The FDR corrected significance values were obtained for the comparison between tumor and normal tissue. In addition, we also calculated the log2 fold change for each individual tumor vs. normal matched tissue using the normalized count values.

### Visualizing coverage along a single repetitive element subfamily consensus

To better examine coverage of repetitive element subfamilies along the full length of the elements we built RepConsensus, an extension of previous efforts to characterize read coverage with respect to a consensus element with added visualization tools [12]. RepConsensus is a package independent of RepEnrich that can be used to visualize coverage of reads along a consensus element. Alignment parameters needed to be more relaxed such that reads containing SNPs can still map to the consensus element and reads that contain adjacent non-repetitive genomic sequence may also map. Consensus elements were downloaded from RepBase.org, including the human-specific L1 element L1Hs. To align reads to the L1Hs consensus we used Bowtie2 local alignment mode (bowtie2 --no-unal -p 16 -N 1 --local -x L1Hs −1 pair1.fastq −2 pair2.fastq -S out.sam). Local alignment mode can soft-clip the reads to allow alignment, which helps align reads that may contain adjacent non-repetitive genomic sequence. The –N 1 option allows for up to one mismatch in the seed sequence, which aids in the mapping of reads containing SNPs different from the consensus. We also build the background distribution of L1 family element genomic instances that map to the L1Hs subfamily consensus using these parameters. This is done to understand the degree with which other highly related subfamilies (such as evolutionarily recent primate-specific L1PA subfamilies) also map to the L1Hs consensus. In addition, we can determine the background distribution of L1 element lengths in the genome. We map all the L1 family genomic instances to L1Hs using the same parameters. Then we calculate the cumulative distribution of L1 genomic instances start and end sites with respect to the length of the L1Hs element. This reveals a preponderance of 5′ truncated elements consistent with what is known about L1 insertions, however, few elements contain 3′ truncations [57]. The information regarding the start and end sites along the L1Hs consensus is important when interpreting RNA-seq alignment to the consensus. To do the analysis of RNA-seq data for prostate cancer, we mapped all the data for tumor and normal matched control to the L1Hs consensus. Then we computed the coverage along the L1Hs consensus using bedtools. The data were normalized by the total mapping reads and the paired calculation of log2 fold change was computed along the length of L1Hs consensus for each individual tumor.

### Investigating the genic vs. intergenic contribution of L1Hs and L1PA RNA-seq transcripts in prostate cancer

To approximate the genic and intergenic contribution of transcripts we examined the reads that mapped uniquely to the genome. We defined L1Hs and L1PA coordinates that overlapped 99% within gene bodies and 99% overlapping with intergenic regions using bedtools and Refseq hg19 gene annotations. Next we computed the coverage for genic L1Hs and L1PA elements and intergenic L1Hs and L1PA elements using bedtools. We summed the coverage for genic L1Hs and L1PA elements and intergenic L1Hs and L1PA elements and then computed the counts per million for these two values without TMM normalization using the total mapping reads. Finally for the paired tumor and normal matched control we computed the log2FC for tumor vs. normal from the normalized log2CPM values.

### Availability of additional files

All data presented in this study was previously published and is publicly available. For detailed summary of samples used see Additional file 2: Table S1. The data is available online through the ENCODE consortium (http://genome.ucsc.edu/ENCODE/). Published datasets are available through the NCBI Gene Expression Omnibus. Oler, A.J. et al. [24] accession number: GSE20309, Canella, D. et al. [15] accession number: GSE18184, and through the European Nucleotide Archive Ren S. et al. [21] accession number: ERP000550.

## Electronic supplementary material

Figure S1 ***RepEnrich*** **read counting strategies**. Examples of the three different read counting strategies tested for use by *RepEnrich*. A) *Total counts*: sums the reads that fall into an individual repetitive element subfamily and allows for multiple counting of reads. B) *Unique counts*: sums the reads that only fall uniquely into a single subfamily of repetitive elements and excludes reads mapping to more than one subfamily. C) *Fractional counts*: sums the reads that fall into each individual repetitive element subfamily and assigns a fraction to reads mapping to more than one subfamily (1/# of repetitive element subfamilies aligned).

Figure S2 **HMM parameters used in the ChIP-seq simulations**. The ChIP-seq simulation was designed to simulate an enriched state over a specified genomic feature and a background state for the remaining regions of a given chromosome. We simulated six conditions, a set of three conditions enriched for L1 LINE, Alu SINE, and SVA retrotransposons and a second set of three conditions where L1 LINE, Alu SINE, and SVA retrotransposons were at background levels for use as input (bottom set). The HMM simulator generates a binding profile along a chromosome reflecting the probability of observing a protein bound to the DNA at every location. By changing the transition state matrix of specified genomic features such that the probability of accessing a high emission state (3.0 +/- σ=0.1) is more likely these features more frequently access the high emission state. Reads are more likely to be randomly sampled from nucleotide positions with high probabilities assigned to them by the HMM. Specified features will tend to yield more reads, or enrichment, due to their more frequent assignment to a high probability state. The remainder of the chromosome will tend to have a background profile, with some narrow regions showing high signal (reflecting normal variability in background signal). Three triplicate samples were generated in each simulation (for more details see materials and methods).

Figure S3 **Genome browser view of simulated data**. In this ChIP-seq simulation L1 LINE retrotransposons were specified to have a transition matrix leading to significant enrichment over background (Figure S5). The reads were generated at 30, 50, and 100 base pairs and were aligned uniquely to the genome using *Bowtie*. The genome browser view shows visually that our method of ChIP-seq simulation may be used to effectively generate enrichment over a specified genomic feature, such as the L1 LINE retrotransposons.

Figure S4 **Comparison of counting strategies performance on Alu enriched simulated ChIP-seq data for human chromosome 19**. A ChIP-seq simulation was conducted such that Alu SINE retrotransposons displayed enrichment with three replicates per condition and 50 bp single-end reads. Since the true coordinates for the reads are known we computed and compared each counting strategy to the true abundance. The average log2CPM read abundances, computed from *RepEnrich* estimated count values using *total*, *unique*, and *fractional* count methods were compared to the true abundance. The solid line indicates y=x, values falling on the line are identical between the estimation and expected. A) The unique count method. B) The total count method. C) The fractional count method. D) Multidimensional scaling (MDS) plot of the Euclidean distances between the average log2CPM values for the *unique*, *total*, *and fraction* estimates and the true abundance average log2CPM values.

Figure S5**Comparison of counting strategy performance over a wide-range of parameters for human chromosome 5**. ChIP-seq simulations were conducted over six conditions (L1, Alu, and SVA retrotransposon enriched samples and corresponding input samples) and three read lengths (35, 50, and 75 base pairs) using two million reads for human chromosome 5. The average abundance, in log2CPM, for each estimate for unique fraction, and total counting methods and the true abundance were computed from the EdgeR TMM normalized counts (see materials and methods). For each simulation R-squared values computed with respect to the true read abundance (R-squared = $1 = \frac{{\sum {{(y - y)}^2}}}{{\sum {{(y - \overline y .)}^2}}}$, x= true abundance, y=estimated abundance) for all repetitive elements and individual classes of repetitive elements. R-squared values less than or equal to zero were aggregated and adjusted to zero (marked by a red circle). B-D) Representative plots of the L1 enriched condition at 35 base pair read length plotted for the unique, fraction, and total counting method respectively. The diagonal line is y=x, for which the R-squared was computed.

Figure S6 **Comparison of counting strategy performance over a wide-range of parameters for human chromosome 10**. R-squared values computed with respect to the expected read abundance for various ChIP-seq simulations on chromosome 10 (Processed identically to figure S5). B-D) Representative plots of the L1 enriched condition at 35 base pair read length plotted for the unique, fraction, and total counting method respectively.

Figure S7 **Comparison of counting strategy differential enrichment analysis predictions for ChIP-seq data simulations over human chromosome 5**. ChIP-seq simulations were conducted over six conditions (L1, Alu, and SVA retrotransposon enriched samples and corresponding input samples) and three read lengths (35, 50, and 75 base pairs) using two million reads for human chromosome 5. We used the bioconductor package EdgeR to conduct differential enrichment analysis for the L1, Alu, and SVA retrotransposon enriched samples compared to input samples. A-I) Display Venn diagrams overlapping the repetitive element subfamilies identified as being significantly differentially enriched (FDR < 0.05) for the various simulations. The benchmark represents the elements identified as significantly different in the analysis of the true abundance, while unique, fraction, and total are the elements identified as significant for the counts obtained by these three count estimates.

Figure S8 **Comparison of counting strategy differential enrichment analysis predictions for ChIP-seq data simulations over human chromosome 10**. ChIP-seq simulations were conducted over six conditions (L1, Alu, and SVA retrotransposon enriched samples and corresponding input samples) and three read lengths (35, 50, and 75 base pairs) using two million reads for human chromosome 10. A-I) Display Venn diagrams overlapping the repetitive element subfamilies identified as being significantly differentially enriched (FDR < 0.05) for the various simulations. The benchmark represents the elements identified as significantly different in the analysis of the true abundance, while unique, fraction, and total are the elements identified as significant for the counts obtained by these three count estimates.

Figure S9 **Comparison of counting strategy differential enrichment analysis predictions for real ChIP-seq data**. A comparison of RNA polymerase II ChIP-seq versus input was conducted on real biological data from ENCODE project for the K562 CML cell-line. We used the bioconductor package EdgeR to conduct differential enrichment analysis of TMM normalized count data for both the Pol II ChIP-seq and input samples (for more details see materials and methods). A) Venn diagram of the overlapping repetitive element sub-families identified as significantly differentially enriched (FDR <0.05) for the unique and fraction counting strategies. Included in the Venn diagram are repetitive elements that are significant in both directions (positive and negative Log2FC) for all classes of repetitive elements including TEs, satellites, and small RNA classes.

Figure S10 Representative genome browser view of ENCODE enrichment tracks. Select RNA Pol II bound repetitive elements identified by RepEnrich to be significantly enriched with respect to input were examined for evidence of enrichment in ENCODE tracks, which are based on the unique mapping alignment. A) Example of a tRNA identified to display enrichment for binding by RNA Pol II, active RNA Pol II-S2, and RNA Pol III by RepEnrich. ENCODE enrichment tracks also display evidence of co-occupancy of RNA Pol II and III at this respective tRNA. **B**) Example of U6 snRNAs identified to display enrichment for binding by RNA Pol II and RNA Pol III by RepEnrich. ENCODE enrichment tracks also display evidence of co-occupancy of RNA Pol II and III at this respective U6 snRNA.

Figure S11 **Pol III promoter-type assignment**. Percent of Pol III bound subfamilies assigned to the different types of Pol III promoters based on the binding activity of the Pol III associated transcription factors. Repetitive elements are assigned to the Type 1/Type 2 class if they have positive enrichment for Bdp1 in more than 50% of the cell lines, and the number of cell lines with positive enrichment for Brf1 is larger than that in which Brf2 or SNAP45 show positive enrichment. Repetitive elements are assigned to the Type 3 class if they have positive enrichment for Bdp1 in more than 50% of the cell lines, and the number of cell lines with positive enrichment for Brf1 is smaller than that in which Brf2 or SNAP45 show positive enrichment. For all other cases, no call is made.

Figure S12 ENCODE RNA Polymerases and differential RNA-seq analysis of SINE, tRNA, and Satellite class elements. Meta-analysis of repetitive element transcriptional activity. The data for RNA-Pol II, active RNA-Polymerase II-S2, RNA-Polymerase III, TFIIIB subunits were visualized using log2FC values from the GLM comparison alongside the log2FC values for RNA-seq differential expression analysis of cytosol PolyA+ vs. PolyA-, nucleus PolyA+ vs. PolyA-, PolyA+ cytosol vs. nucleus, PolyA- cytosol vs. nucleus, and total RNA nucleoplasm vs. nucleolus. In the plot comparisons that were significant, with an FDR<0.05, are represented using red (up) or blue (down). For the ChIP-seq log2FCs negative values were replaced by zero (no change). A) SINE class elements B) tRNA class elements C) Satellite class elements.

Figure S13 ENCODE RNA Polymerases and differential RNA-seq analysis of DNA, LINE, and LTR class elements. Meta-analysis of repetitive element transcriptional activity. The data for RNA-Pol II, active RNA-Pol II-S2, RNA-Pol III, TFIIIB subunits were visualized using log2FC values from the GLM comparison alongside the log2FC values for RNA-seq differential expression analysis of cytosol PolyA+ vs. PolyA-, nucleus PolyA+ vs. PolyA-, PolyA+ cytosol vs. nucleus, PolyA- cytosol vs. nucleus, and total RNA nucleoplasm vs. nucleolus. In the plot comparisons that were significant, with an FDR<0.05, are represented using red (up) or blue (down). For the ChIP-seq log2FCs negative values were replaced by zero (no change). A) DNA class elements B) LINE class elements C) LTR class elements.

Figure S14 **Summary of RNA polymerase II enrichment to LTR retrotransposons in ENCODE cell-lines**. The log2 fold change of Pol II ChIP with respect to input for LTR retrotransposons that were significant (FDR <0.05) in at least one cell-line are plotted for two cancer (K562 and Hela), one transformed (GM12878), and three normal (Pbde, Huvec, and IMR90) cell-lines. Hierarchical clustering was done based on the log2 fold change using Euclidean metrics.

Figure S15 Estimation of genic vs. intergenic contribution of L1PA and L1Hs elements. L1Hs and L1PA elements were annotated as genic (overlapping with Refseq genes) or intergenic (not overlapping with Refseq genes). The coverage of genic and intergenic L1Hs and L1PA genomic instances was computed from the unique mapping reads of prostate tumor samples and normal samples. The sum of counts for the genic and intergenic L1Hs and L1PA elements was then computed for normal and tumor samples. The count was normalized by total mapping reads and the log2FC of tumor versus normal was computed for each individual. Genic and intergenic L1Hs and L1PA elements were similarly expressed in tumor samples expressing high levels of L1 retrotransposons (group 1).

Figure S16 **Example of homology between RE L1PA subfamilies**. Clustal Omega multiple sequence alignment of L1PA2, L1PA3, L1PA4, L1PA5, and L1PA6 retrotransposons [59]. L1PA retrotransposons are the most recent family of primate specific L1 LINE retrotransposons and show a high degree of homology due to their evolutionary recent divergence from a common L1 retrotransposon.

Figure S17 **Effect of read length on repetitive element subfamily read assignment**. Analysis of simulated data by *RepEnrich* reveals key features and biases associated with estimating repetitive element enrichment in high-throughput sequencing data. Read length affects the ability to distinguish between highly homologous repetitive element subfamilies. A simulated ChIP-seq sample was trimmed from 100 base pairs to 50 and 30 base pairs. The identical sample sequenced with different read lengths were analyzed by *RepEnrich*. The reads from each sample that were categorically assigned to repetitive elements was then examined for the number of distinctive repetitive element subfamilies the reads aligned. A) Log10 proportion of reads plotted as a function of number of repetitive element subfamilies reads aligned. B) Reads analyzed by *RepEnrich* were binned and determined as falling into a unique repetitive element subfamily, 2- 10 repetitive element subfamilies, or >10 subfamilies.

Table S1 Description of publically available datasets used in this study. Set 1–7 refers to how the datasets were grouped into separate GLM models that were used in our analysis

## Abbreviations

RTEs: Retrotransposable elements
LTR: Long terminal repeat
LINEs: Long interspersed nuclear elements
SINEs: Short interspersed nuclear elements
Pol: RNA polymerase
CAGE: Cap analysis gene expression
TSS: Transcriptional start site
HMM: Hidden markov model
CPM: Counts per million mapping reads
rRNA: Ribosomal RNA
CML: Chronic myelogenous leukemia
PBDE: Peripheral blood-derived erythroblast cells
GLM: Generalized linear model
HERV: Human endogenous retrovirus
TBP: TATA-box binding protein
DHS: DNase I hypersensitive sites
ALS: Amyotrophic lateral sclerosis
MS: Multiple sclerosis
ESC: Embryonic stem cells.
