# Supplementary material for: Transcriptional landscape of repetitive elements in normal and cancer human cells
Source: BMC Genomics. 2014 Jul 11;15:583. doi: 10.1186/1471-2164-15-583 (PMC4122776; doi:10.1186/1471-2164-15-583)
Supplement: Supplementary file 1 — RepEnrich read counting strategies. Examples of the three different read counting strategies tested for use by RepEnrich. A) Total counts: sums the reads that fall into an individual repetitive element subfamily and allows for multiple counting of reads. B) Unique counts: sums the reads that only fall uniquely into a single subfamily of repetitive elements and excludes reads mapping to more than one subfamily. C) Fractional counts: sums the reads that fall into each individual repetitive element subfamily and assigns a fraction to reads mapping to more than one subfamily (1/# of repetitive element subfamilies aligned). [file 12864_2014_6313_MOESM1_ESM.pdf]

A

**Repeat subfamily**	**AluY**	**AluJ**	**L1H**	**L1PA2**
read1	1	0	0	0
read2	1	1	0	0
read3	0	0	1	1
read4	0	0	0	1
**Total counts**	**2**	**1**	**1**	**2**

B

**Repeat subfamily**	**AluY**	**AluJ**	**L1H**	**L1PA2**
read1	1	0	0	0
~~read2~~	~~1~~	~~1~~	0	0
~~read3~~	0	0	~~1~~	~~1~~
read4	0	0	0	1
**Unique counts**	**1**	**0**	**0**	**1**

C

**Repeat subfamily**	**AluY**	**AluJ**	**L1H**	**L1PA2**
read1	1	0	0	0
read2	1/2	1/2	0	0
read3	0	0	1/2	1/2
read4	0	0	0	1
**Fractional counts**	**3/2**	**1/2**	**1/2**	**3/2**

Figure S1
