# Supplementary material for: Transcriptional landscape of repetitive elements in normal and cancer human cells
Source: BMC Genomics. 2014 Jul 11;15:583. doi: 10.1186/1471-2164-15-583 (PMC4122776; doi:10.1186/1471-2164-15-583)
Supplement: Supplementary file 2 — HMM parameters used in the ChIP-seq simulations. The ChIP-seq simulation was designed to simulate an enriched state over a specified genomic feature and a background state for the remaining regions of a given chromosome. We simulated six conditions, a set of three conditions enriched for L1 LINE, Alu SINE, and SVA retrotransposons and a second set of three conditions where L1 LINE, Alu SINE, and SVA retrotransposons were at background levels for use as input (bottom set). The HMM simulator generates a binding profile along a chromosome reflecting the probability of observing a protein bound to the DNA at every location. By changing the transition state matrix of specified genomic features such that the probability of accessing a high emission state (3.0 +/- σ=0.1) is more likely these features more frequently access the high emission state. Reads are more likely to be randomly sampled from nucleotide positions with high probabilities assigned to them by the HMM. Specified features will tend to yield more reads, or enrichment, due to their more frequent assignment to a high probability state. The remainder of the chromosome will tend to have a background profile, with some narrow regions showing high signal (reflecting normal variability in background signal). Three triplicate samples were generated in each simulation (for more details see materials and methods). [file 12864_2014_6313_MOESM2_ESM.pdf]

Emissions:
high = ↑ = 3.0 +/- σ = 0.1
low = ↓ = 0.5+/- σ=0.1

L1 enriched sample 1 (n=3)

L1↑:

	↓	↑
↓	0.50	0.50
↑	0.05	0.95

else↓:

	↓	↑
↓	0.95	0.05
↑	0.6	0.4

Input sample 1 (n=3)

L1↑:

	↓	↑
↓	0.95	0.05
↑	0.6	0.4

else↓:

	↓	↑
↓	0.95	0.05
↑	0.6	0.4

chr 5, $2*10^6$ reads
chr 10, $2*10^6$ reads
chr 19, $2*10^7$ reads

Alu enriched sample 2 (n=3)

Alu↑:

	↓	↑
↓	0.50	0.50
↑	0.05	0.95

else↓:

	↓	↑
↓	0.95	0.05
↑	0.6	0.4

Input sample 2 (n=3)

Alu↑:

	↓	↑
↓	0.95	0.05
↑	0.6	0.4

else↓:

	↓	↑
↓	0.95	0.05
↑	0.6	0.4

chr 5, $2*10^6$ reads
chr 10, $2*10^6$ reads
chr 19, $2*10^7$ reads

SVA enriched sample 3 (n=3)

L1↑:

	↓	↑
↓	0.50	0.50
↑	0.05	0.95

else↓:

	↓	↑
↓	0.95	0.05
↑	0.6	0.4

Input sample 3 (n=3)

L1↑:

	↓	↑
↓	0.95	0.05
↑	0.6	0.4

else↓:

	↓	↑
↓	0.95	0.05
↑	0.6	0.4

chr 5, $2*10^6$ reads
chr 10, $2*10^6$ reads

Figure S2
