# Supplementary figures and images for: Transcriptional landscape of repetitive elements in normal and cancer human cells
Source: BMC Genomics. 2014 Jul 11;15:583. doi: 10.1186/1471-2164-15-583 (PMC4122776; doi:10.1186/1471-2164-15-583)

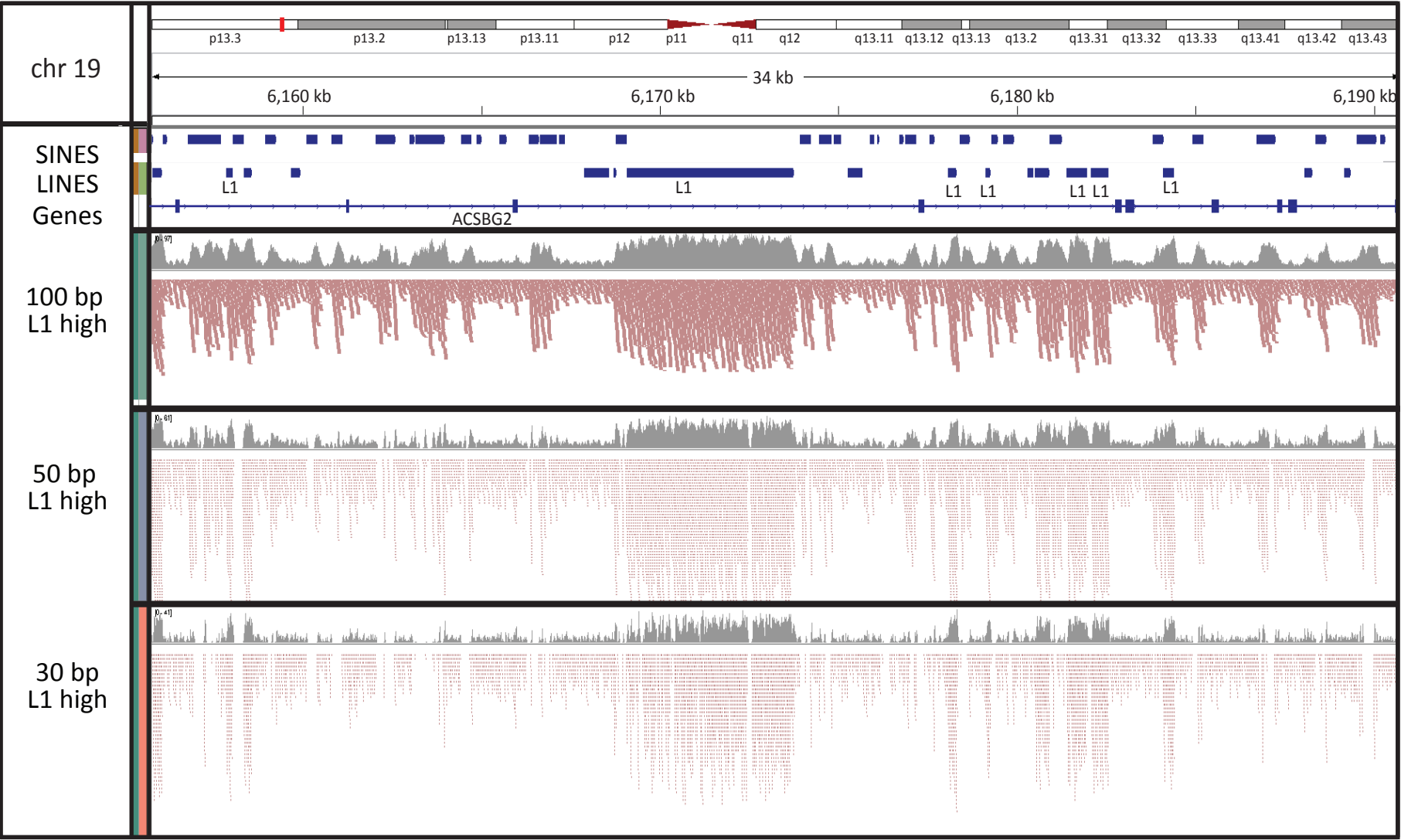

Figure S3

Supplement: Supplementary file 3 — Genome browser view of simulated data. In this ChIP-seq simulation L1 LINE retrotransposons were specified to have a transition matrix leading to significant enrichment over background (Figure S5). The reads were generated at 30, 50, and 100 base pairs and were aligned uniquely to the genome using Bowtie. The genome browser view shows visually that our method of ChIP-seq simulation may be used to effectively generate enrichment over a specified genomic feature, such as the L1 LINE retrotransposons. [file 12864_2014_6313_MOESM3_ESM.pdf]

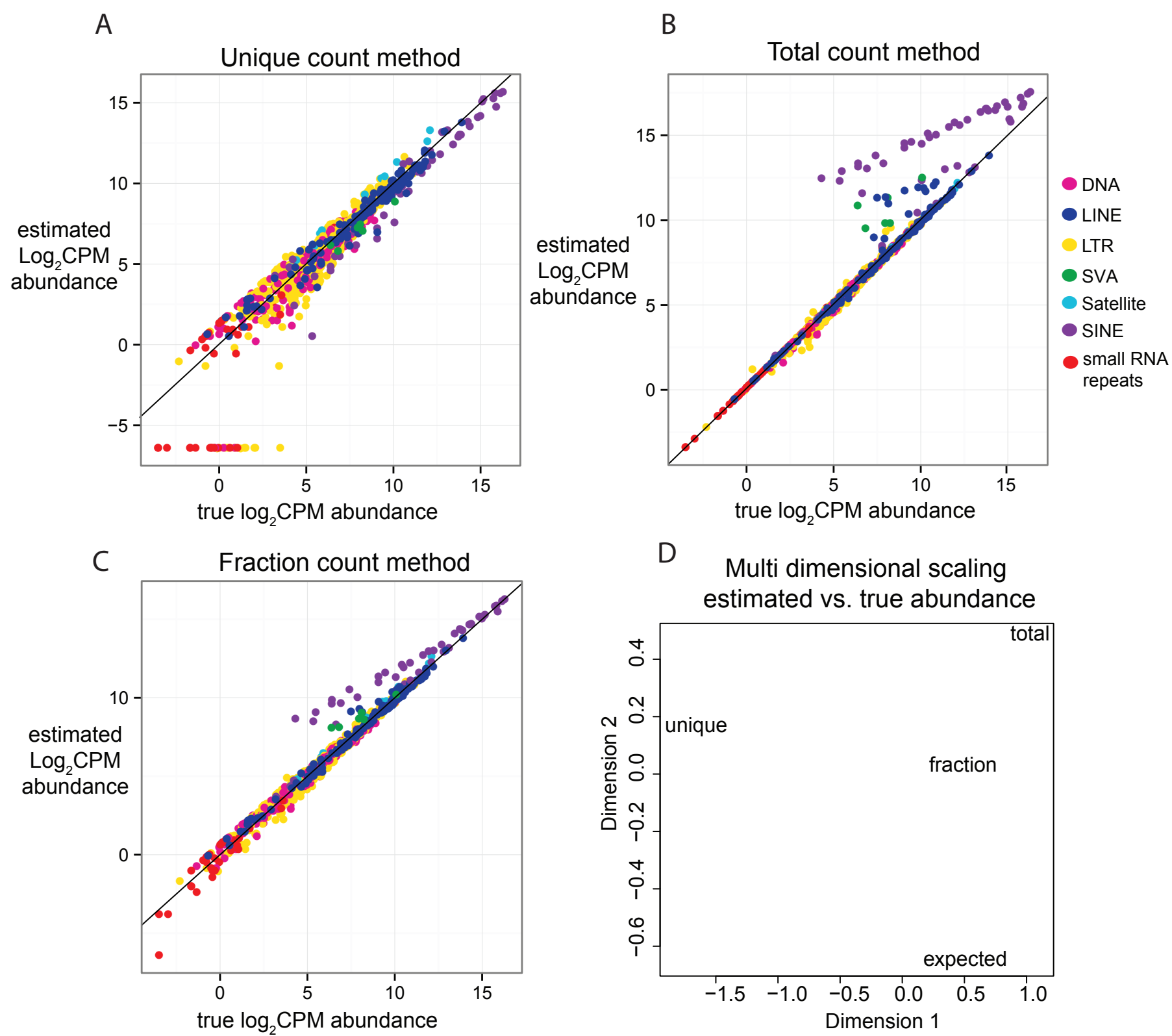

Figure S4

Supplement: Supplementary file 4 — Comparison of counting strategies performance on Alu enriched simulated ChIP-seq data for human chromosome 19. A ChIP-seq simulation was conducted such that Alu SINE retrotransposons displayed enrichment with three replicates per condition and 50 bp single-end reads. Since the true coordinates for the reads are known we computed and compared each counting strategy to the true abundance. The average log2CPM read abundances, computed from RepEnrich estimated count values using total, unique, and fractional count methods were compared to the true abundance. The solid line indicates y=x, values falling on the line are identical between the estimation and expected. A) The unique count method. B) The total count method. C) The fractional count method. D) Multidimensional scaling (MDS) plot of the Euclidean distances between the average log2CPM values for the unique, total, and fraction estimates and the true abundance average log2CPM values. [file 12864_2014_6313_MOESM4_ESM.pdf]

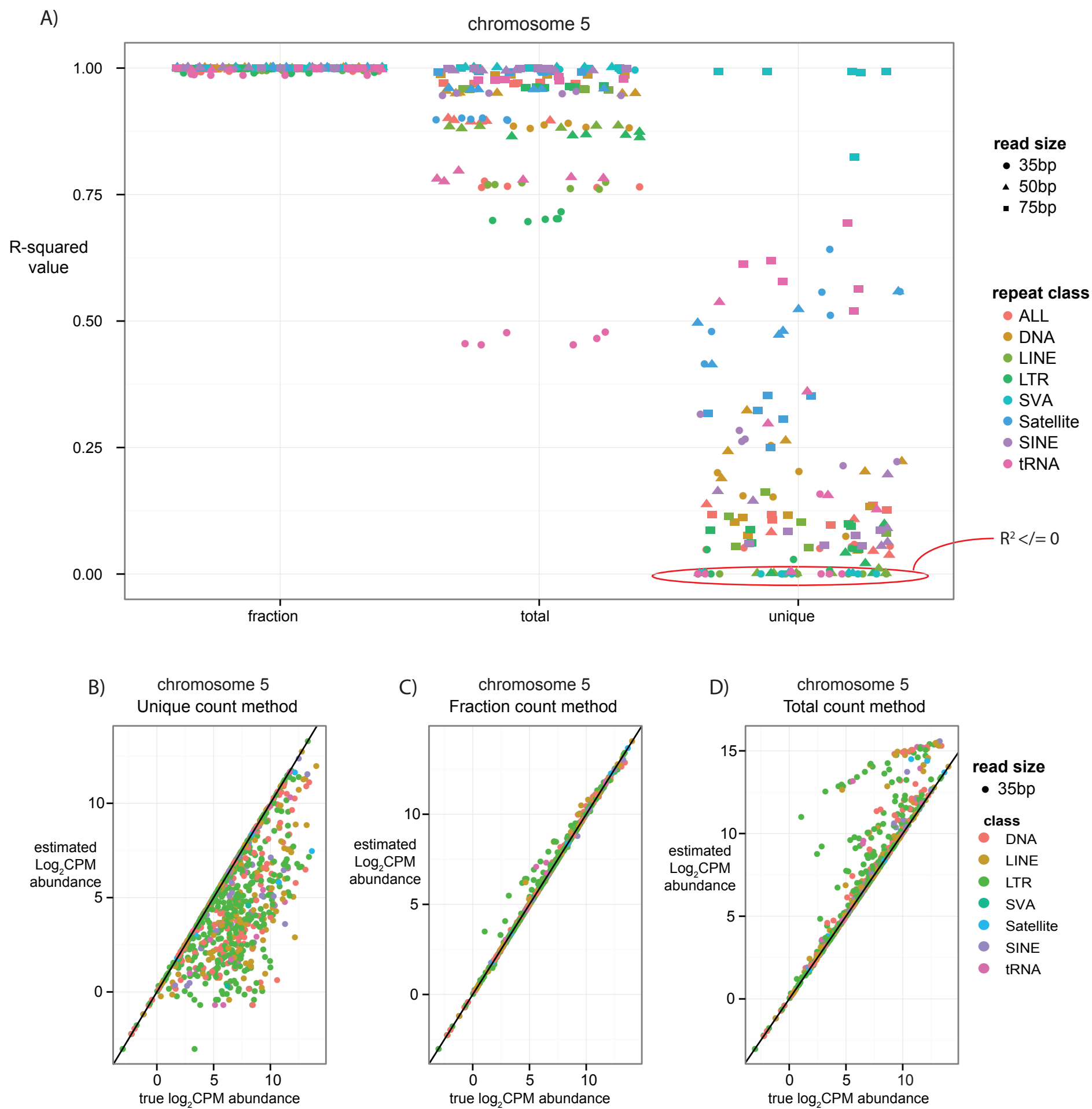

Figure S5

Supplement: Supplementary file 5 — Comparison of counting strategy performance over a wide-range of parameters for human chromosome 5. ChIP-seq simulations were conducted over six conditions (L1, Alu, and SVA retrotransposon enriched samples and corresponding input samples) and three read lengths (35, 50, and 75 base pairs) using two million reads for human chromosome 5. The average abundance, in log2CPM, for each estimate for unique fraction, and total counting methods and the true abundance were computed from the EdgeR TMM normalized counts (see materials and methods). For each simulation R-squared values computed with respect to the true read abundance (R-squared = $1 = \frac{{\sum {{(y - y)}^2}}}{{\sum {{(y - \overline y .)}^2}}}$1=∑(y−y)2∑(y−y¯.)2, x= true abundance, y=estimated abundance) for all repetitive elements and individual classes of repetitive elements. R-squared values less than or equal to zero were aggregated and adjusted to zero (marked by a red circle). B-D) Representative plots of the L1 enriched condition at 35 base pair read length plotted for the unique, fraction, and total counting method respectively. The diagonal line is y=x, for which the R-squared was computed. [file 12864_2014_6313_MOESM5_ESM.pdf]

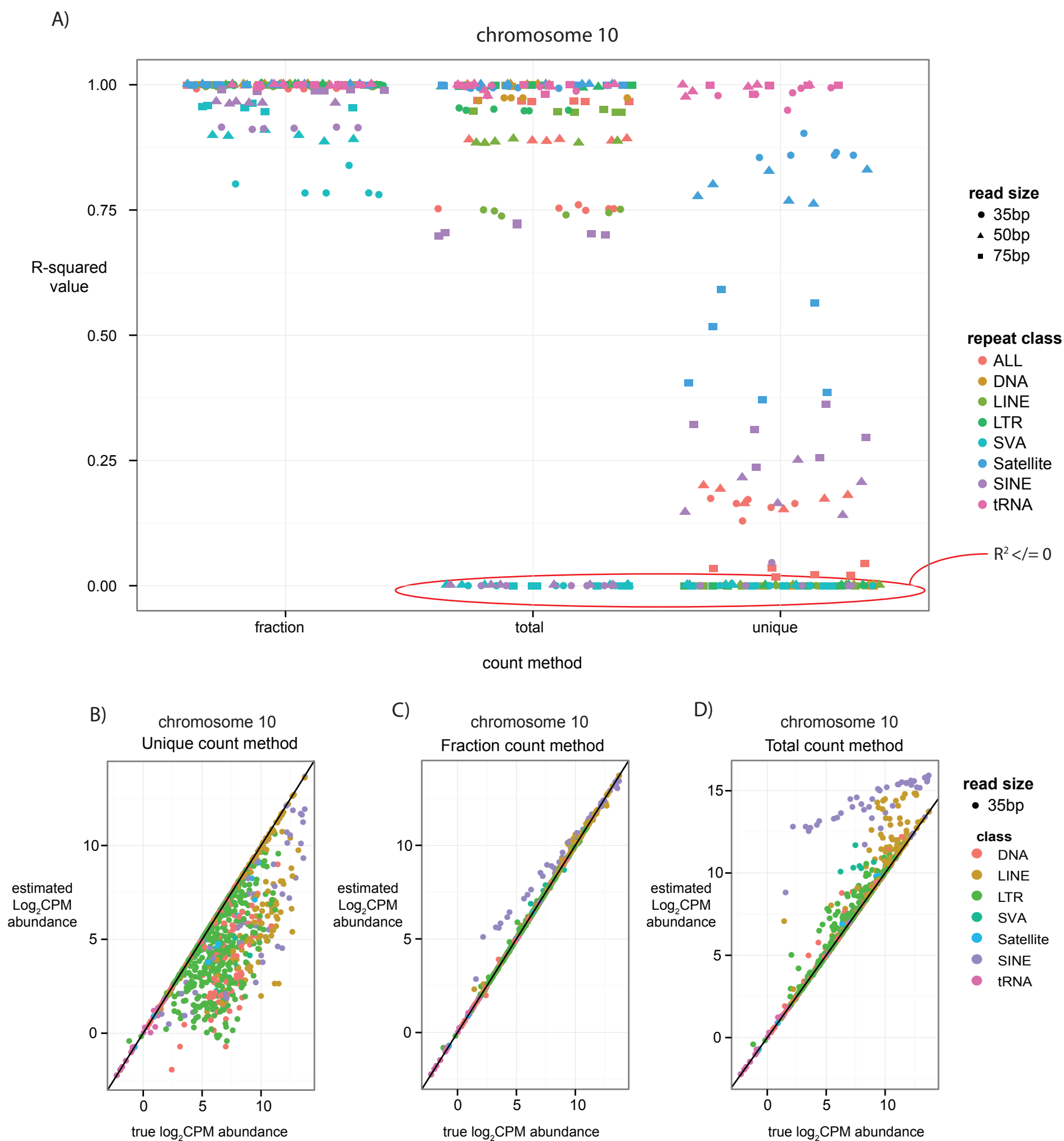

Figure S6

Supplement: Supplementary file 6 — Comparison of counting strategy performance over a wide-range of parameters for human chromosome 10. R-squared values computed with respect to the expected read abundance for various ChIP-seq simulations on chromosome 10 (Processed identically to figure S5). B-D) Representative plots of the L1 enriched condition at 35 base pair read length plotted for the unique, fraction, and total counting method respectively. [file 12864_2014_6313_MOESM6_ESM.pdf]

A)

K562 Pol II ChIP, FDR<0.05

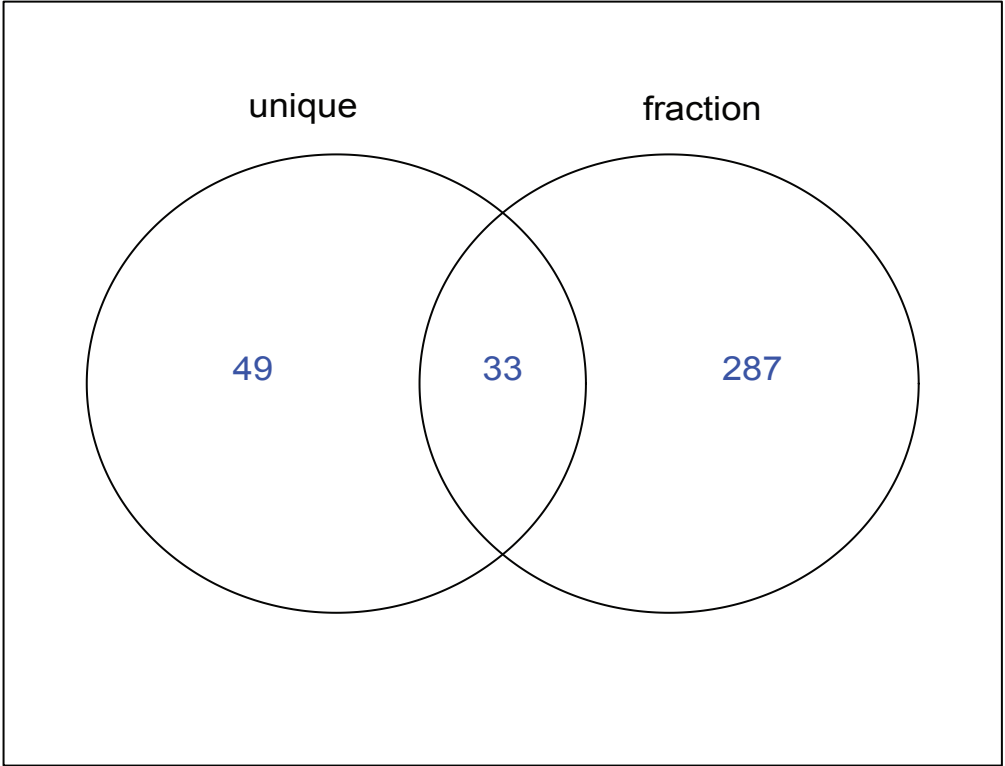

Figure S9

Supplement: Supplementary file 9 — Comparison of counting strategy differential enrichment analysis predictions for real ChIP-seq data. A comparison of RNA polymerase II ChIP-seq versus input was conducted on real biological data from ENCODE project for the K562 CML cell-line. We used the bioconductor package EdgeR to conduct differential enrichment analysis of TMM normalized count data for both the Pol II ChIP-seq and input samples (for more details see materials and methods). A) Venn diagram of the overlapping repetitive element sub-families identified as significantly differentially enriched (FDR <0.05) for the unique and fraction counting strategies. Included in the Venn diagram are repetitive elements that are significant in both directions (positive and negative Log2FC) for all classes of repetitive elements including TEs, satellites, and small RNA classes. [file 12864_2014_6313_MOESM9_ESM.pdf]

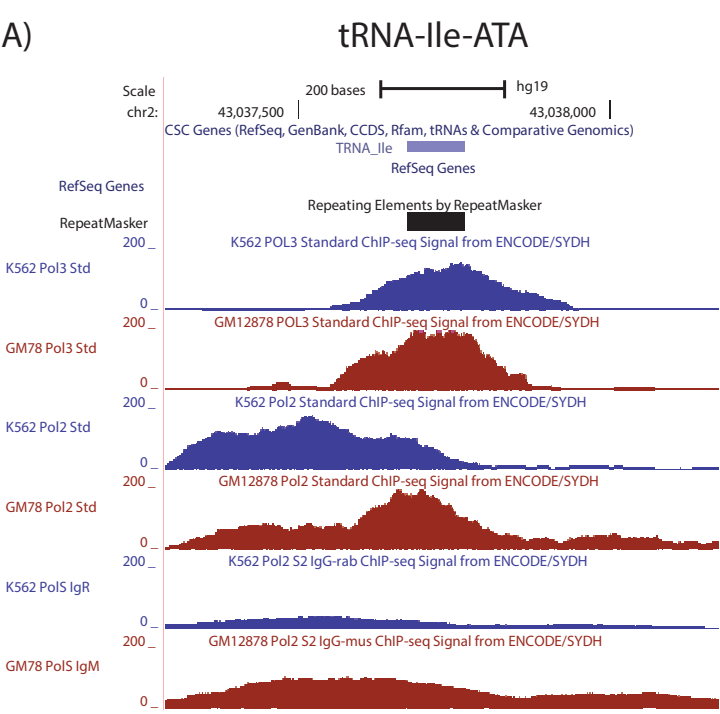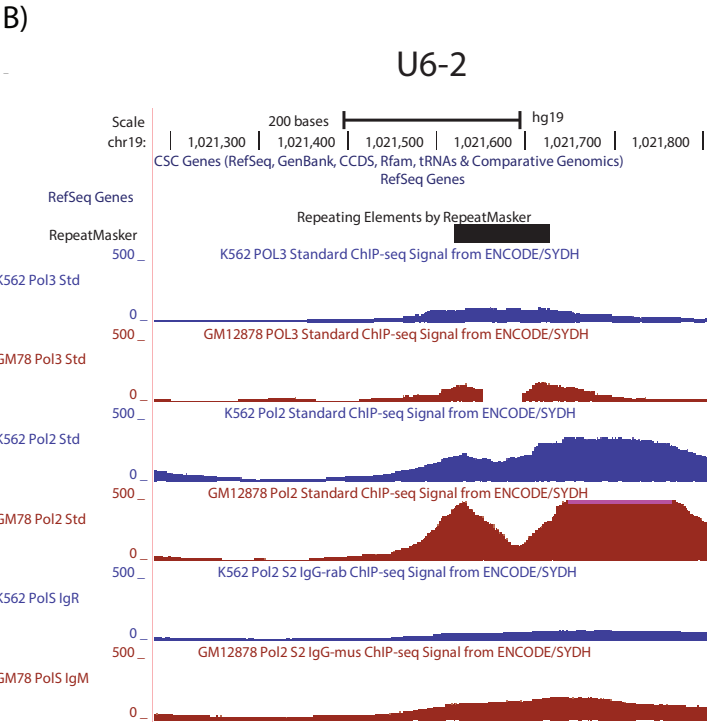

Figure S10

Supplement: Supplementary file 10 — Representative genome browser view of ENCODE enrichment tracks. Select RNA Pol II bound repetitive elements identified by RepEnrich to be significantly enriched with respect to input were examined for evidence of enrichment in ENCODE tracks, which are based on the unique mapping alignment. A) Example of a tRNA identified to display enrichment for binding by RNA Pol II, active RNA Pol II-S2, and RNA Pol III by RepEnrich. ENCODE enrichment tracks also display evidence of co-occupancy of RNA Pol II and III at this respective tRNA. B) Example of U6 snRNAs identified to display enrichment for binding by RNA Pol II and RNA Pol III by RepEnrich. ENCODE enrichment tracks also display evidence of co-occupancy of RNA Pol II and III at this respective U6 snRNA. [file 12864_2014_6313_MOESM10_ESM.pdf]

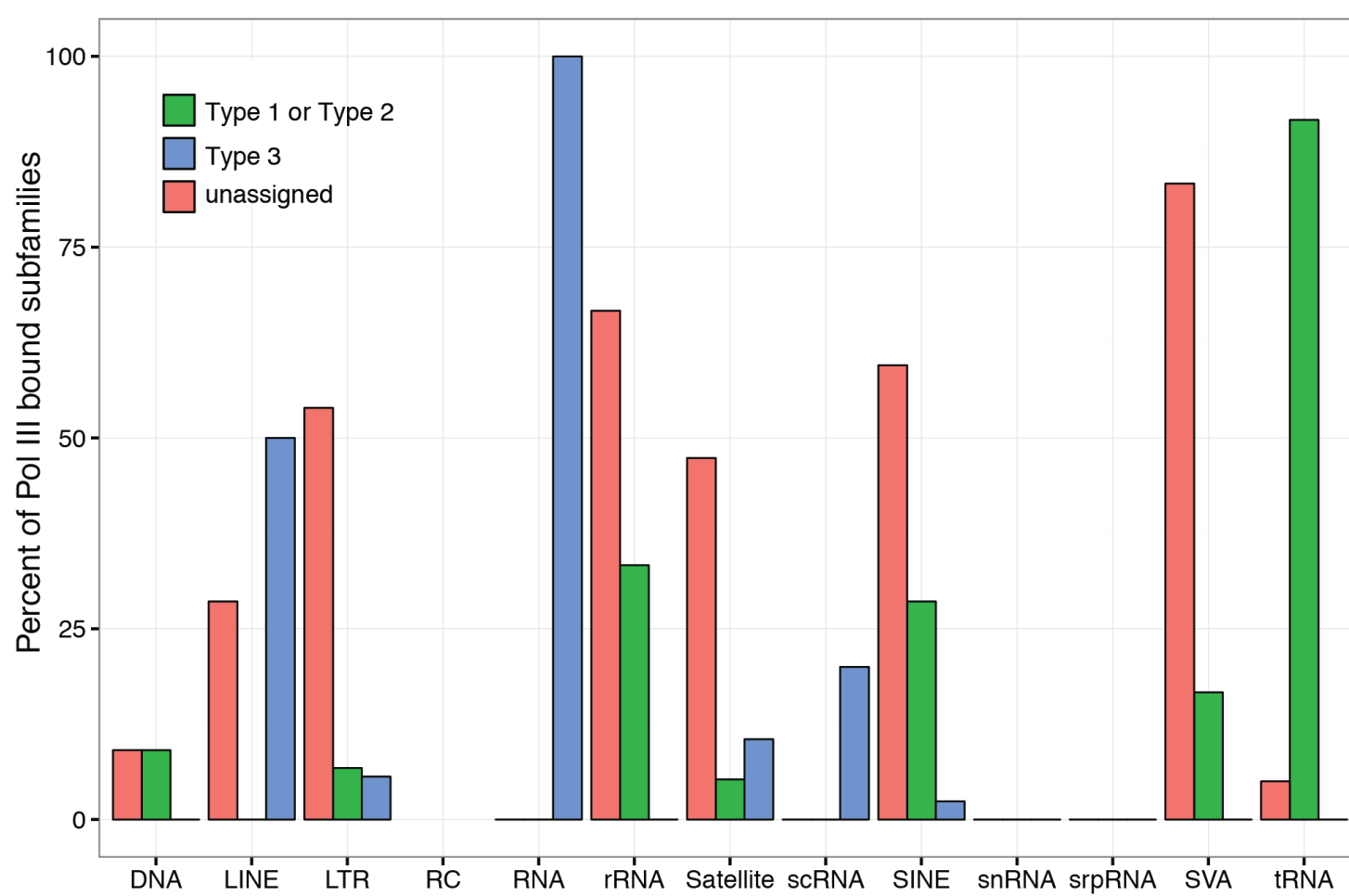

Figure S11

Supplement: Supplementary file 11 — Pol III promoter-type assignment. Percent of Pol III bound subfamilies assigned to the different types of Pol III promoters based on the binding activity of the Pol III associated transcription factors. Repetitive elements are assigned to the Type 1/Type 2 class if they have positive enrichment for Bdp1 in more than 50% of the cell lines, and the number of cell lines with positive enrichment for Brf1 is larger than that in which Brf2 or SNAP45 show positive enrichment. Repetitive elements are assigned to the Type 3 class if they have positive enrichment for Bdp1 in more than 50% of the cell lines, and the number of cell lines with positive enrichment for Brf1 is smaller than that in which Brf2 or SNAP45 show positive enrichment. For all other cases, no call is made. [file 12864_2014_6313_MOESM11_ESM.pdf]

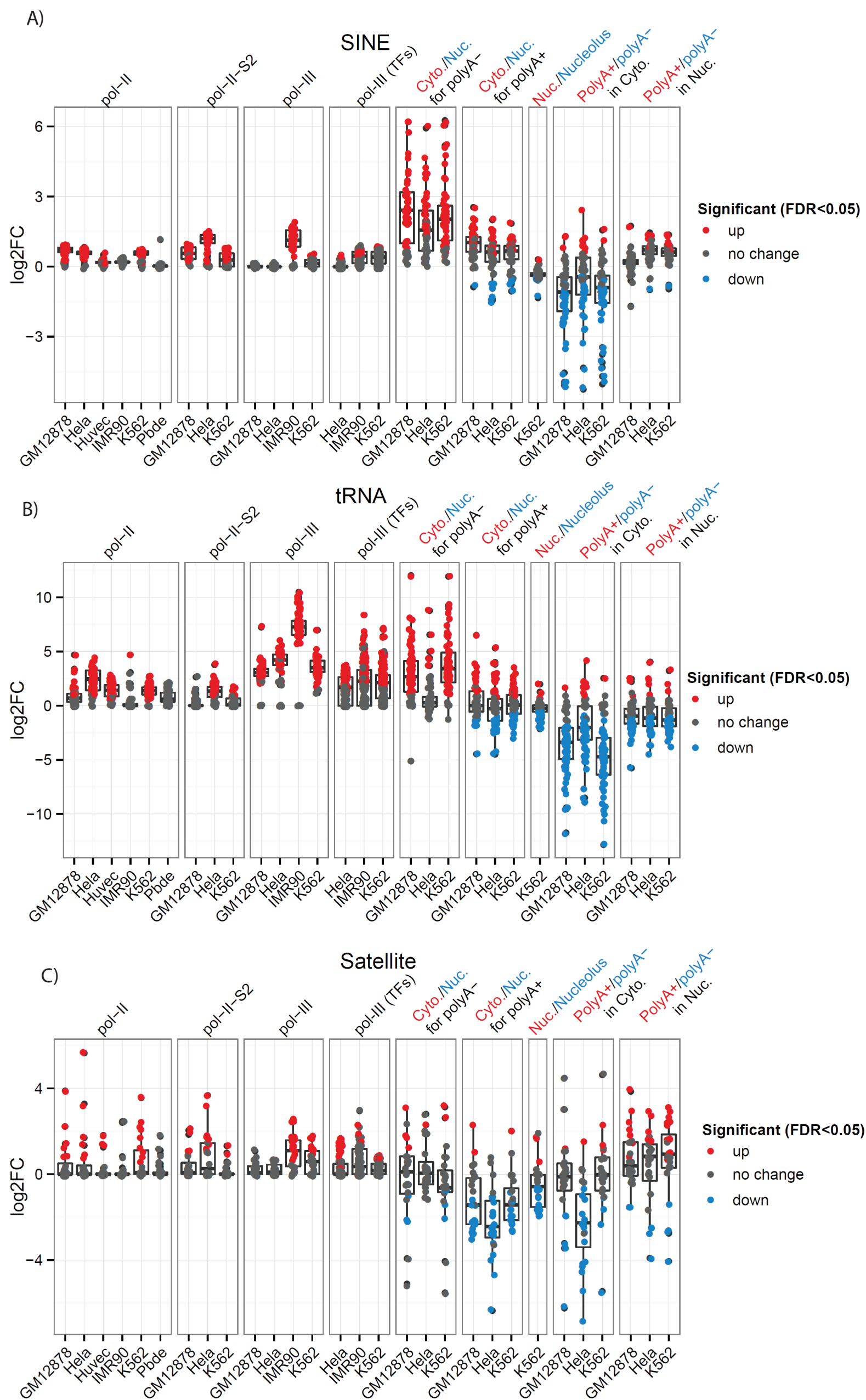

Figure S12

Supplement: Supplementary file 12 — ENCODE RNA Polymerases and differential RNA-seq analysis of SINE, tRNA, and Satellite class elements. Meta-analysis of repetitive element transcriptional activity. The data for RNA-Pol II, active RNA-Polymerase II-S2, RNA-Polymerase III, TFIIIB subunits were visualized using log2FC values from the GLM comparison alongside the log2FC values for RNA-seq differential expression analysis of cytosol PolyA+ vs. PolyA-, nucleus PolyA+ vs. PolyA-, PolyA+ cytosol vs. nucleus, PolyA- cytosol vs. nucleus, and total RNA nucleoplasm vs. nucleolus. In the plot comparisons that were significant, with an FDR<0.05, are represented using red (up) or blue (down). For the ChIP-seq log2FCs negative values were replaced by zero (no change). A) SINE class elements B) tRNA class elements C) Satellite class elements. [file 12864_2014_6313_MOESM12_ESM.pdf]

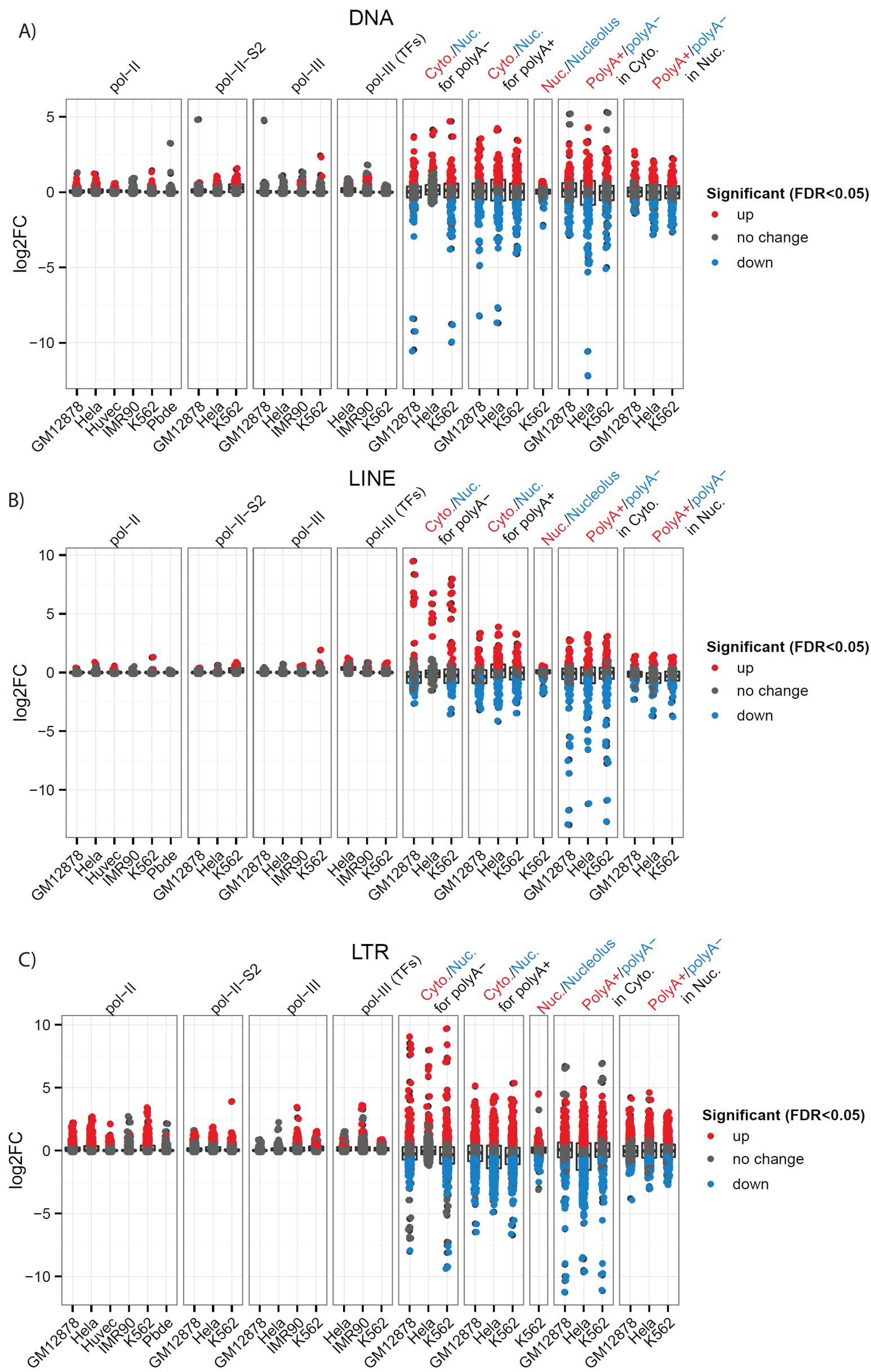

Figure S13

Supplement: Supplementary file 13 — ENCODE RNA Polymerases and differential RNA-seq analysis of DNA, LINE, and LTR class elements. Meta-analysis of repetitive element transcriptional activity. The data for RNA-Pol II, active RNA-Pol II-S2, RNA-Pol III, TFIIIB subunits were visualized using log2FC values from the GLM comparison alongside the log2FC values for RNA-seq differential expression analysis of cytosol PolyA+ vs. PolyA-, nucleus PolyA+ vs. PolyA-, PolyA+ cytosol vs. nucleus, PolyA- cytosol vs. nucleus, and total RNA nucleoplasm vs. nucleolus. In the plot comparisons that were significant, with an FDR<0.05, are represented using red (up) or blue (down). For the ChIP-seq log2FCs negative values were replaced by zero (no change). A) DNA class elements B) LINE class elements C) LTR class elements. [file 12864_2014_6313_MOESM13_ESM.pdf]

LTR class elements  
FDR<0.05 in at least one Pol-II ChIP-seq

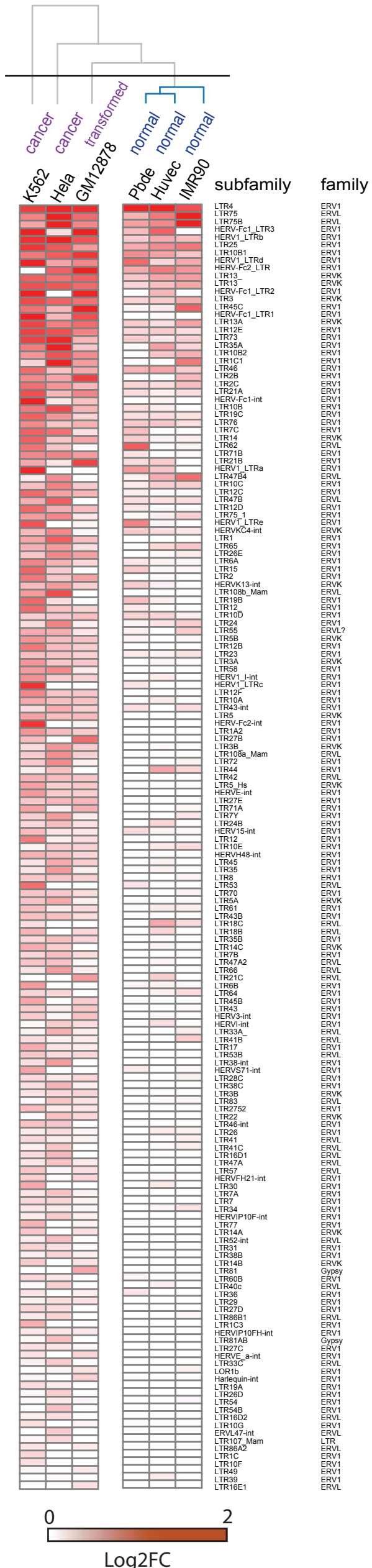

Figure S14

Supplement: Supplementary file 14 — Summary of RNA polymerase II enrichment to LTR retrotransposons in ENCODE cell-lines. The log2 fold change of Pol II ChIP with respect to input for LTR retrotransposons that were significant (FDR <0.05) in at least one cell-line are plotted for two cancer (K562 and Hela), one transformed (GM12878), and three normal (Pbde, Huvec, and IMR90) cell-lines. Hierarchical clustering was done based on the log2 fold change using Euclidean metrics. [file 12864_2014_6313_MOESM14_ESM.pdf]

Genic vs. intergenic L1PA and L1Hs unique

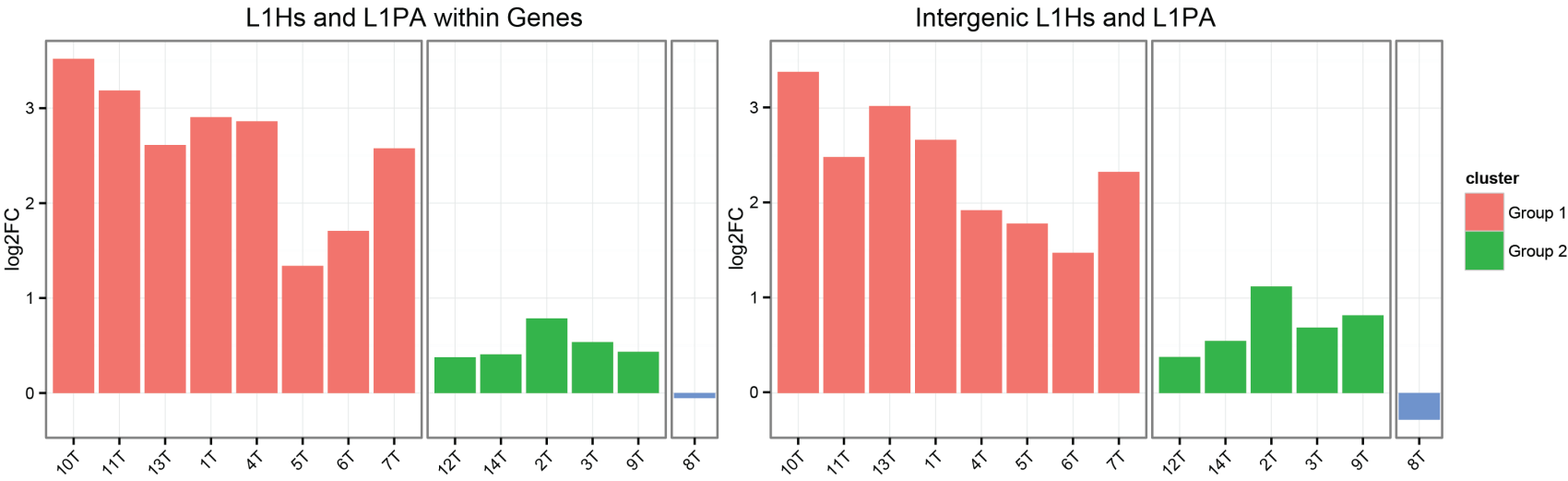

Figure S15

Supplement: Supplementary file 15 — Estimation of genic vs. intergenic contribution of L1PA and L1Hs elements. L1Hs and L1PA elements were annotated as genic (overlapping with Refseq genes) or intergenic (not overlapping with Refseq genes). The coverage of genic and intergenic L1Hs and L1PA genomic instances was computed from the unique mapping reads of prostate tumor samples and normal samples. The sum of counts for the genic and intergenic L1Hs and L1PA elements was then computed for normal and tumor samples. The count was normalized by total mapping reads and the log2FC of tumor versus normal was computed for each individual. Genic and intergenic L1Hs and L1PA elements were similarly expressed in tumor samples expressing high levels of L1 retrotransposons (group 1). [file 12864_2014_6313_MOESM15_ESM.pdf]

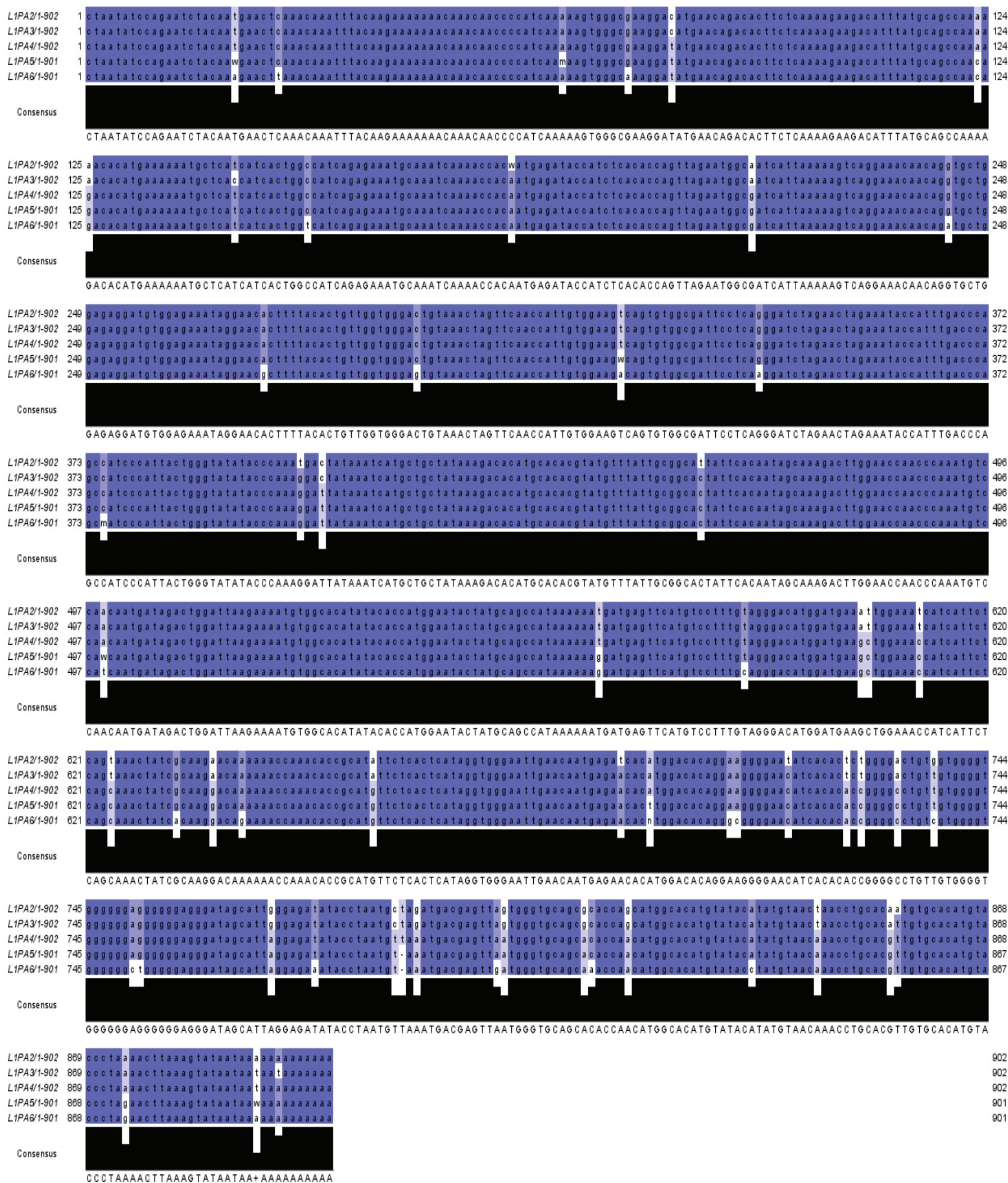

Figure S16

Supplement: Supplementary file 16 — Example of homology between RE L1PA subfamilies. Clustal Omega multiple sequence alignment of L1PA2, L1PA3, L1PA4, L1PA5, and L1PA6 retrotransposons [59]. L1PA retrotransposons are the most recent family of primate specific L1 LINE retrotransposons and show a high degree of homology due to their evolutionary recent divergence from a common L1 retrotransposon. [file 12864_2014_6313_MOESM16_ESM.pdf]

A) Effect of read length

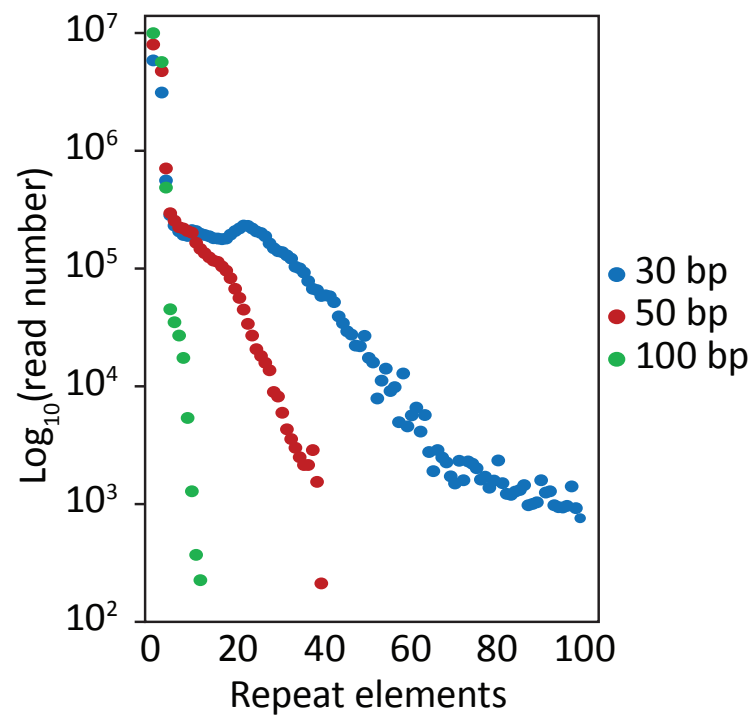

B) % Mapping to multiple elements

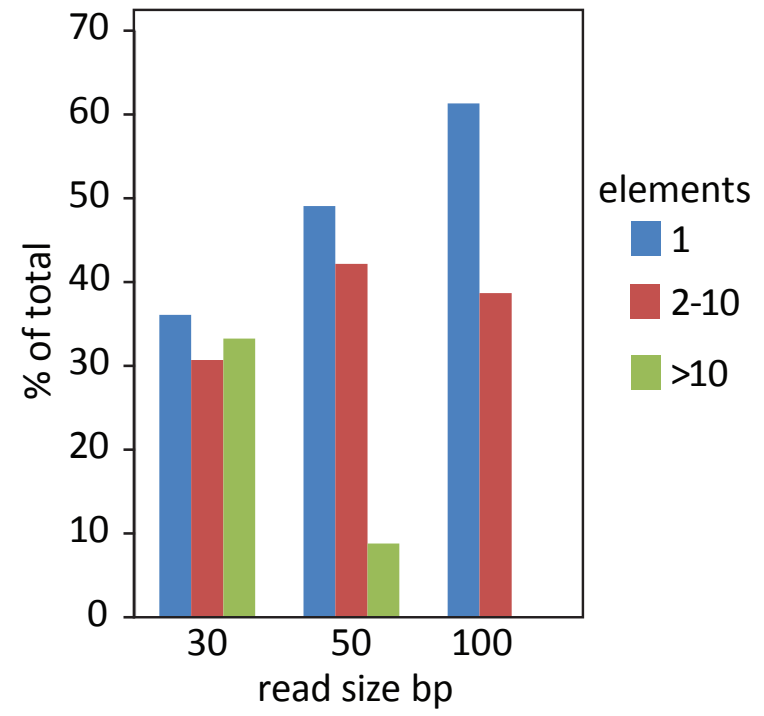

Figure S17

Supplement: Supplementary file 17 — Effect of read length on repetitive element subfamily read assignment. Analysis of simulated data by RepEnrich reveals key features and biases associated with estimating repetitive element enrichment in high-throughput sequencing data. Read length affects the ability to distinguish between highly homologous repetitive element subfamilies. A simulated ChIP-seq sample was trimmed from 100 base pairs to 50 and 30 base pairs. The identical sample sequenced with different read lengths were analyzed by RepEnrich. The reads from each sample that were categorically assigned to repetitive elements was then examined for the number of distinctive repetitive element subfamilies the reads aligned. A) Log10 proportion of reads plotted as a function of number of repetitive element subfamilies reads aligned. B) Reads analyzed by RepEnrich were binned and determined as falling into a unique repetitive element subfamily, 2- 10 repetitive element subfamilies, or >10 subfamilies. [file 12864_2014_6313_MOESM17_ESM.pdf]
