# Supplementary material for: Transcriptional landscape of repetitive elements in normal and cancer human cells
Source: BMC Genomics. 2014 Jul 11;15:583. doi: 10.1186/1471-2164-15-583 (PMC4122776; doi:10.1186/1471-2164-15-583)
Supplement: Supplementary file 8 — Comparison of counting strategy differential enrichment analysis predictions for ChIP-seq data simulations over human chromosome 10. ChIP-seq simulations were conducted over six conditions (L1, Alu, and SVA retrotransposon enriched samples and corresponding input samples) and three read lengths (35, 50, and 75 base pairs) using two million reads for human chromosome 10. A-I) Display Venn diagrams overlapping the repetitive element subfamilies identified as being significantly differentially enriched (FDR < 0.05) for the various simulations. The benchmark represents the elements identified as significantly different in the analysis of the true abundance, while unique, fraction, and total are the elements identified as significant for the counts obtained by these three count estimates. [file 12864_2014_6313_MOESM8_ESM.pdf]

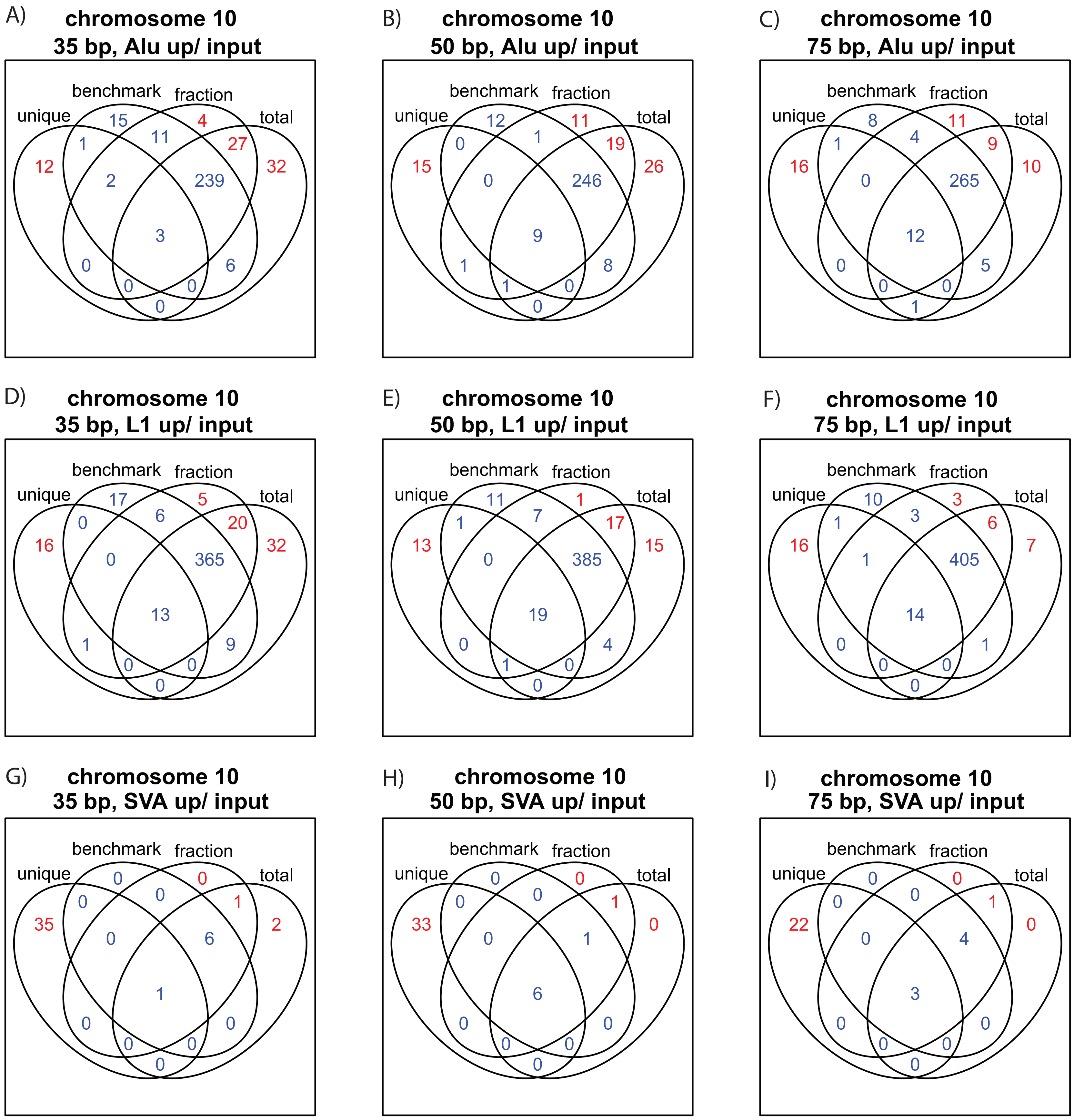
A) chromosome 10
35 bp, Alu up/ input
benchmark fraction unique total
15 4 1 11 27 12 32 2 239 3 0 6 0 0 0
B) chromosome 10
50 bp, Alu up/ input
benchmark fraction unique total
12 11 0 1 19 15 26 0 246 9 1 8 1 0 0
C) chromosome 10
75 bp, Alu up/ input
benchmark fraction unique total
8 11 1 4 9 16 10 0 265 12 0 5 0 0 1
D) chromosome 10
35 bp, L1 up/ input
benchmark fraction unique total
17 5 0 6 20 16 32 0 365 13 1 9 0 0 0
E) chromosome 10
50 bp, L1 up/ input
benchmark fraction unique total
11 1 1 7 17 13 15 0 385 19 0 4 1 0 0
F) chromosome 10
75 bp, L1 up/ input
benchmark fraction unique total
10 3 1 3 6 16 7 1 405 14 0 1 0 0 0
G) chromosome 10
35 bp, SVA up/ input
benchmark fraction unique total
0 0 0 0 1 35 2 0 6 1 0 0 0 0 0
H) chromosome 10
50 bp, SVA up/ input
benchmark fraction unique total
0 0 0 0 1 33 0 0 1 6 0 0 0 0 0
I) chromosome 10
75 bp, SVA up/ input
benchmark fraction unique total
0 0 0 0 1 22 0 0 4 3 0 0 0 0 0

Figure S8
