# Supplementary material for: Transcriptional landscape of repetitive elements in normal and cancer human cells
Source: BMC Genomics. 2014 Jul 11;15:583. doi: 10.1186/1471-2164-15-583 (PMC4122776; doi:10.1186/1471-2164-15-583)
Supplement: Supplementary file 7 — Comparison of counting strategy differential enrichment analysis predictions for ChIP-seq data simulations over human chromosome 5. ChIP-seq simulations were conducted over six conditions (L1, Alu, and SVA retrotransposon enriched samples and corresponding input samples) and three read lengths (35, 50, and 75 base pairs) using two million reads for human chromosome 5. We used the bioconductor package EdgeR to conduct differential enrichment analysis for the L1, Alu, and SVA retrotransposon enriched samples compared to input samples. A-I) Display Venn diagrams overlapping the repetitive element subfamilies identified as being significantly differentially enriched (FDR < 0.05) for the various simulations. The benchmark represents the elements identified as significantly different in the analysis of the true abundance, while unique, fraction, and total are the elements identified as significant for the counts obtained by these three count estimates. [file 12864_2014_6313_MOESM7_ESM.pdf]

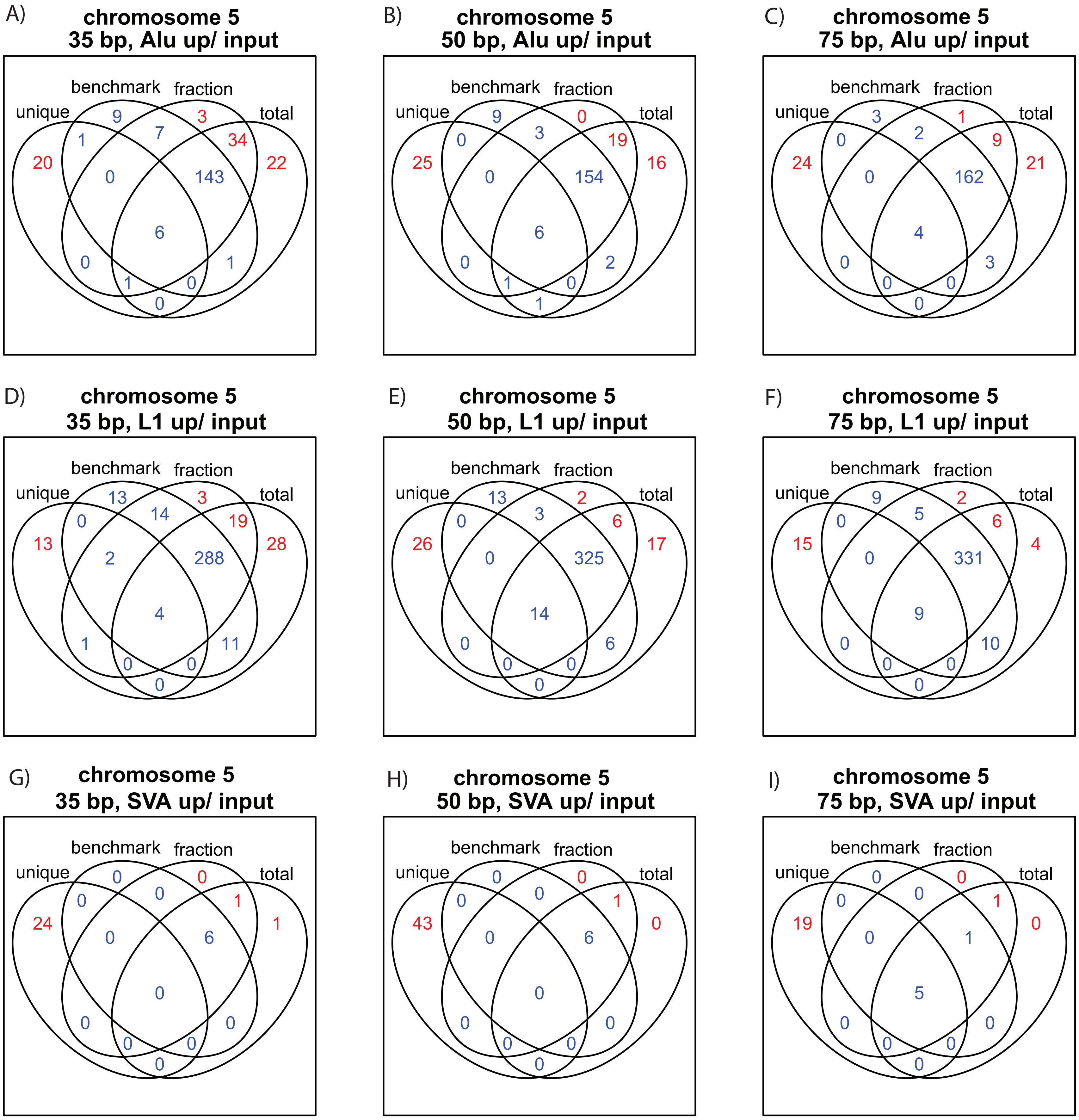
A)
chromosome 5
35 bp, Alu up/ input
benchmark
fraction
unique
total
9
3
1
7
34
20
0
143
22
6
0
1
1
0
0
B)
chromosome 5
50 bp, Alu up/ input
benchmark
fraction
unique
total
9
0
0
3
19
25
0
154
16
6
0
2
1
0
1
C)
chromosome 5
75 bp, Alu up/ input
benchmark
fraction
unique
total
3
1
0
2
9
24
0
162
21
4
0
3
0
0
0
D)
chromosome 5
35 bp, L1 up/ input
benchmark
fraction
unique
total
13
3
0
14
19
13
2
288
28
4
1
11
0
0
0
E)
chromosome 5
50 bp, L1 up/ input
benchmark
fraction
unique
total
13
2
0
3
6
26
0
325
17
14
0
6
0
0
0
F)
chromosome 5
75 bp, L1 up/ input
benchmark
fraction
unique
total
9
2
0
5
6
15
0
331
4
9
0
10
0
0
0
G)
chromosome 5
35 bp, SVA up/ input
benchmark
fraction
unique
total
0
0
0
0
1
24
0
6
1
0
0
0
0
0
0
H)
chromosome 5
50 bp, SVA up/ input
benchmark
fraction
unique
total
0
0
0
0
1
43
0
6
0
0
0
0
0
0
0
I)
chromosome 5
75 bp, SVA up/ input
benchmark
fraction
unique
total
0
0
0
0
1
19
0
1
0
5
0
0
0
0
0

Figure S7
